# In Silico Identification of Potential Thyroid Hormone System Disruptors among Chemicals in Human Serum and Chemicals with a High Exposure Index

**DOI:** 10.1021/acs.est.1c07762

**Published:** 2022-05-13

**Authors:** Elena Dracheva, Ulf Norinder, Patrik Rydén, Josefin Engelhardt, Jana M. Weiss, Patrik L. Andersson

**Affiliations:** † Department of Chemistry, Umeå University, KB.E6, Linnaeus väg 6, SE-901 87 Umeå, Sweden; ‡ Department of Computer and Systems Sciences, Stockholm University, Box 7003, SE-164 07 Kista, Sweden; § Department of Mathematics and Mathematical Statistics, Umeå University, MIT.E.351, SE-901 87 Umeå, Sweden; ∥ Department of Environmental Science, Stockholm University, SE-11418 Stockholm, Sweden

**Keywords:** conformal prediction, environmental health, endocrine disruption, QSAR

## Abstract

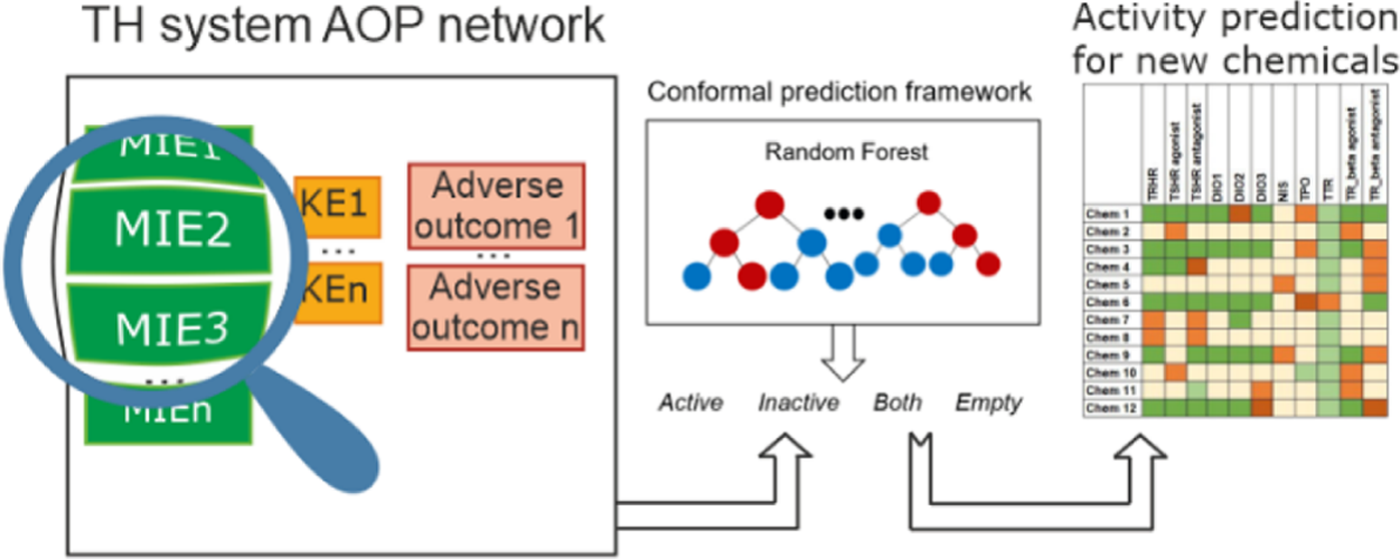

Data on toxic effects are at large missing the prevailing understanding of the risks of industrial chemicals. Thyroid hormone (TH) system disruption includes interferences of the life cycle of the thyroid hormones and may occur in various organs. In the current study, high-throughput screening data available for 14 putative molecular initiating events of adverse outcome pathways, related to disruption of the TH system, were used to develop 19 in silico models for identification of potential thyroid hormone system-disrupting chemicals. The conformal prediction framework with the underlying Random Forest was used as a wrapper for the models allowing for setting the desired confidence level and controlling the error rate of predictions. The trained models were then applied to two different databases: (i) an in-house database comprising xenobiotics identified in human blood and ii) currently used chemicals registered in the Swedish Product Register, which have been predicted to have a high exposure index to consumers. The application of these models showed that among currently used chemicals, fewer were overall predicted as active compared to chemicals identified in human blood. Chemicals of specific concern for TH disruption were identified from both databases based on their predicted activity.

## Abstract 2

Identification of potential thyroid hormone system-disrupting chemicals with confidence among human health- and exposure-relevant chemicals.

## Introduction

Humans are constantly exposed to manmade chemicals through various sources including diet, consumer products, and building materials, and these chemicals often lack data on environmental and human health effects. Lack of hazard information is a problem in understanding the causes of various diseases, including disturbance of the endocrine system. In the past years, the attention on xenobiotics as potential endocrine disruptors has increased and much research focus within the field has been directed toward disruption of the estrogen and androgen mediated pathways. Another critical endocrine related pathway is the thyroid hormone (TH) system. Thyroid hormones are known to play a critical role in a number of tissues at various developmental stages and to be involved in processes such as regulation of energy utilization, cell cycle control via binding to nuclear receptors,^1−3^ metabolism, the female reproductive system,^4^ as well as development and maintenance of the nervous system.^5,6^ THs influence the brain in all life stages, but in particular, they are of great importance in the prenatal period during early stages of brain development.^7^ Disruption induced by environmental contaminants and industrial chemicals of the TH system is covered in the U.S. EPA Endocrine Disruptor Screening Program (EDSP) along with the estrogen and androgen-related systems.^8,9^

The Hypothalamus–pituitary–thyroid (HPT) axis controls the thyroid hormone synthesis via several mechanisms, thus involving many molecular interactions.^10,11^ In brief, thyroid synthesis is regulated via a negative feedback loop in the HPT axis. Thyrotropin-releasing hormone (TRH) secreted in the hypothalamus binds to the TRH receptor (TRHR) in the pituitary and stimulates the secretion of thyroid-stimulating hormone (TSH),^12^ which in turn binds to the TSH receptor (TSHR) in the thyroid gland, stimulating TH production. Sodium/iodide symporter (NIS) delivers I^–^ into the thyroid cells^13^ and thyroperoxidase (TPO) catalyzes the iodination of tyrosine residues to form thyroglobulin, the precursor of TH.^14,15^ The synthesized THs are then transported in the blood with various transporters including Transthyretin (TTR) and in the target tissues, where three Iodothyronine Deiodinases (DIO1,2,3) mediate the activation of TH.^10^ In the target cells, THs act on nuclear thyroid hormone receptors (TRs) which influence gene expression.^16^ Thyroid hormone disrupting chemicals (THDCs) can modulate any of the above described key mechanisms of the TH system.^11,17^ Receptors related to xenobiotic metabolism such as the aryl hydrocarbon receptor (AhR), constitutive androstane receptor (CAR), pregnane X receptor (PXR) and peroxisome proliferator-activated receptors (PPARs) are also included as molecular targets for thyroid hormone system disruption as they regulate the expression of phase I and II metabolic enzymes which in turn can affect TH catabolism and clearance and lead to changing THs circulating in the organism.^11,18^

Adverse outcome pathway (AOP) frameworks^19^ are means to organize sequences of molecular and cellular events leading to an adverse effect. An AOP starts with a molecular initiating event (MIE) at a molecular target, then proceeds through one or several key events at a higher level of biological organization to an in vivo-observed adverse outcome on the organism level (measured in animal studies).^20^ Over the past years, extensive research has revealed a number of potential MIEs in the TH system pathway,^11^ and the number grows with increasing understanding of the system. Recently, Noyes et al.^11^ reviewed AOPs related to the TH system and high-throughput in vitro assays available to monitor well-known and putative MIEs. As of today, 26 putative MIEs belonging to 7 regulation mechanisms are known or suspected to be involved in regulation of the TH system. THDCs may interact with one MIE or a number of them^18^ and with that as a basis, we reviewed currently available in vitro data for thyroid-related MIEs.

A large number of existing and newly synthesized compounds could disturb the endocrine system, and it is critical to identify these to implement regulations. First-tier in silico methods are means to assist prioritization of chemicals and reduce the number of in vitro and in vivo tests needed for a more thorough risk assessment. (Quantitative) structure–activity relationship models [(Q)SARs] are in silico models allowing screening of large chemical inventories based on quantitative data (QSAR) or qualitative data (SAR) where the potency of chemicals to induce an MIE are related to their structural and physico-chemical properties. Attempts have earlier been made to develop first-tier predictive tools to identify chemicals of concern for their potency to modulate the endocrine system, either focusing on one or several compound classes or targeting a limited number of protein targets related to different hormone systems.^21−23^ Recently, a battery of QSAR models for some of the thyroid-specific MIEs was developed with several classical machine learning methods.^24^

In the current study, our first aim was to develop first-tier predictive QSAR models able to identify potential THDCs with confidence using the Conformal Prediction (CP) framework with the underlying Random Forest. Models were built for all MIEs related to the TH system as recently described by Noyes et al.,^11^ where data for model development is available. These MIEs include both those directly involved in the TH system and those involved in metabolism that may affect levels of THs or TDCs. Second, we applied the developed models to identify and prioritize potential THDCs in two lists of chemicals: i) an in-house database comprised of xenobiotics identified in human blood (Human Blood Database (HBDB), 419 chemicals), and ii) currently used chemicals registered in the Swedish Product Register (SE-PR) which have been predicted to have a high exposure index to consumers based on usage pattern and tonnage (937 chemicals) (see Materials and Methods). Data from developed models on compounds’ predicted interactions with molecular targets in the TH system will aid in the prioritization of further and more detailed toxicological investigations.

## Materials and Methods

### Datasets and Data Curation

Data suitable for modeling were collected for 14 MIEs of which several included both agonistic and antagonistic modes of action yielding in a total of 19 datasets (Table 1). Datasets on eight MIEs (TRHR, TSHR, TR-beta, CAR, PXR, AhR, PPARD, and PPARD) with qualitative outcomes (“active” and “inactive”) were collected from the publicly available database Tox21.^25,26^ The remaining six MIEs datasets (NIS, TPO, DIO1, DIO2, DIO3, and TTR) were retrieved from the open scientific literature and among these the curated data on TTR binding was obtained from previous studies in our group.^27,28^ The TTR dataset included mainly halogenated compounds and/or contained more than one aromatic ring.

**Table 1:** Datasets Used for Model Development^a^

	MIE^b^	mode of action	number of active compounds (% in the set)	number of inactive compounds	source
thyroid-specific	Hypothalamic-pituitary feedback				
	TRHR	antagonist	49 (0.07)	6653	Tox21^c^
	TSHR	agonist	268 (4.2)	6121	Tox21
		antagonist	211 (3.2)	6289	Tox21
	TPO	inhibition	292 (29.6)	696	(30)
	NIS	inhibition	39 (19.2)	164	(32)
	Serum thyroid hormone transport				
	TTR	binding	88 (39.6)	134	(27)
	Thyroid receptor activation				
	TR-beta	agonist	34 (0.5)	6566	Tox21
		antagonist	319 (6)	5003	Tox21
	Thyroid hormones metabolism and excretion				
	DIO1	inhibition	204 (11.8)	1518	(31)
	DIO2	inhibition	275 (16)	1446	(31)
	DIO3	inhibition	293 (17)	1428	(31)
					
general	CAR	agonist	863 (13.7)	5438	Tox21
		antagonist	144 (2.9)	4677	Tox21
	PXR	agonist	1506 (25.1)	4480	Tox21
	AhR	agonist	748 (11.8)	5591	Tox21
	PPARD	agonist	86 (1.4)	5957	Tox21
		antagonist	63 (1.08)	5754	Tox21
	PPARG	agonist	194 (3.1)	6110	Tox21
		antagonist	344 (6.1)	5285	Tox21

a Numbers of active and inactive compounds are stated after the curation procedure

b TRHR—thyrotropin-releasing hormone receptor; TSHR—thyroid-stimulating hormone receptor; TPO—thyroperoxidase; NIS—sodium/iodide symporter; TTR—transthyretin; TR-beta—thyroid receptor beta; DIO1, DIO2, DIO3—iodothyronine deiodinases 1, 2, 3; CAR—constitutive androstane receptor; PXR—pregnane X receptor; AhR—aryl hydrocarbon receptor; PPARD—peroxisome proliferator-activated receptor delta; and PPARG—peroxisome proliferator-activated receptor gamma.

c Tox21 data was taken from refs (25) and (26).

All gathered datasets were subjected to a standardization and curation procedure, which are essential for modeling. ChemAxon Standardizer version 20.3.0^29^ with options “Remove explicit hydrogens”, “Strip salts”, “Remove solvents”, “Aromatize”, “Tautomerize”, and “Clean 2D” was used to standardize compounds in all datasets. While the 14 Tox21 datasets had defined compound activity, the datasets obtained from the literature needed to be processed as described in the Supporting Information.

All datasets were curated with a workflow developed in KNIME Analytical Software^33^ (version 4.1.2). During the curation process, inorganic compounds and mixtures of organic components were removed from the dataset along with records where activity was stated as “inconclusive”. Duplicate structures were handled according to the concordance of the activity value and compounds with inconsistent activity were omitted.

### Classification

The aim was to derive models that successfully could be used to predict chemical compounds as either active or inactive. Here we apply CP with an underlying Random Forest procedure to build predictive QSAR models. 119 RDKit chemical descriptors were calculated, and the models were developed in Python3 using packages for cheminformatics (“rdkit”),^34^ machine learning (“scikit-learn”), and CP (“nonconformist”).

**Figure 1:**
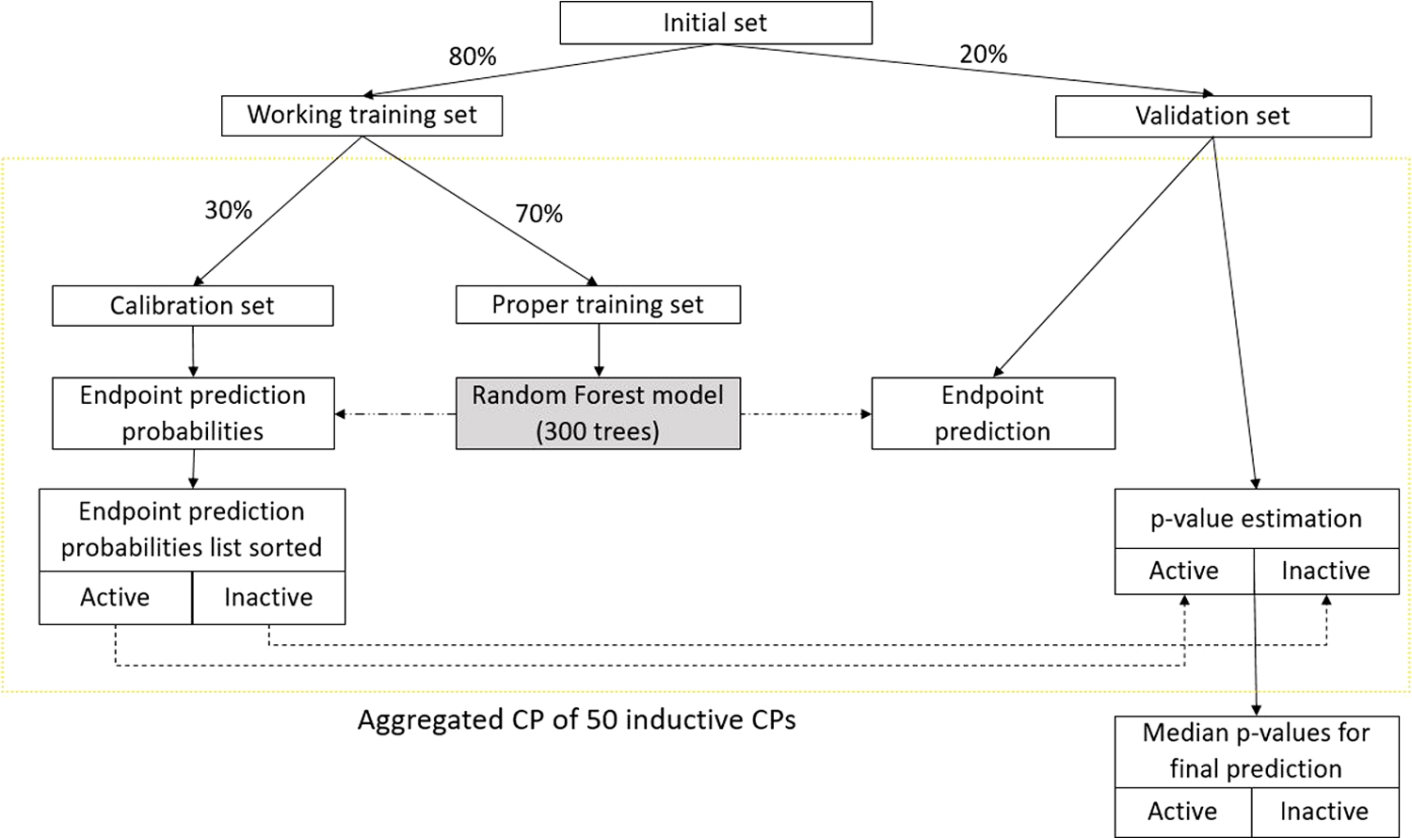
Conformal prediction (CP) framework. After the separation of the validation set (20%), we aggregate 50 CP models built on different random subsets of 70% of the working training set and calibrated on the remaining 30%.

The framework of CP is based on the mathematical proof by Vovk et al.^35^ Here the training set is split into a true training set and a calibration set, where the latter serves to determine the nonconformity measure, that is it shows how dissimilar a chemical is from other chemicals already seen by the model. In contrast to classical supervised methods, in which the compound is predicted as either “active” or “inactive”, CP yields one of four possible prediction regions for a compound: “active”, “inactive”, “both”, or “empty”. The first two predictions are single-label predictions while a compound predicted as “both” cannot be assigned a single label due to the lack of information used to derive the model. Compounds outside the applicability domain of the model are assigned to the empty set. The predictions depend on the user-defined Significance Level (SL), choosing a low SL often results in many compounds predicted as “both” and fewer single-label predictions while a higher SL forces the model to make single-label predictions, and as a result, fewer compounds will be predicted as both.^36−38^ Compounds can be sorted based on their decidability score,^39^ that is the difference between p-values for the active and the inactive class, which is useful when the aim is to select a set of compounds for further studies. The CP framework applied in the current project is summarized in Figure 1.

A random Forest using the default settings, apart from the number of trees that was set to 300 based on the previous experience, was used as the underlying machine learning method in CP, to build predictive QSAR models. For each model, we set aside 20% of the initial set for validation. The remaining 80% were split into a training set (70%) and a calibration set (30%). All splits were carried out using stratified random sampling, which preserves the percentage of active chemicals for each class. The resulting p-values for compounds being predicted were computed with an aggregated model by taking the median of p-values for each class yielded by the 50 individual CPs. Aggregated models for each MIE were validated on the corresponding validation set.

### Model Performance Estimation

CP models are commonly evaluated using the concepts of validity and efficiency. Validity is the percentage of correctly classified chemicals, where the “both” prediction is always considered correct because it contains both predicted classes, and efficiency states the number of single-label predictions, regardless of whether they are correct or not. If the CP model is well-calibrated, the set of predicted labels it produces for a compound contains the true activity value with a guaranteed probability of the set confidence level.

When applying the models, high efficiency is desirable, and the correctness of predictions also has to be considered. For this reason, we combine CP-specific measurements, that is validity and efficiency, with the traditional measurements of classification model performance. True positive rate (TPR), false positive rate (FPR), negative predictive value (NPV) and true discovery rates for *active* and *both* prediction regions (TDR.A and TDR.B, respectively) based on the validation set compounds were calculated. The TPR, FPR, NPV, TDR.A, and TDR.B were estimated as follows
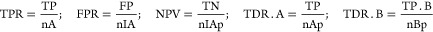
where TP, FP, and TN denote the number of true positives (predicting an active compound as active), false positives (predicting an inactive compound as active), and true negatives (predicting an inactive compound as inactive), respectively; *nA* and *nIA* denote the number of active and inactive compounds in the validation set, respectively; TP.B denotes the number of active compounds in the *both* set; nAp, nBp, and nIAp refer to the number of compounds predicted in *active*, *both*, and *inactive* sets, respectively. Note that neither TPR nor FPR is affected by compounds predicted as *both* or *empty*. The performance of each model was investigated with SLs 0.01, 0.02, 0.05, 0.1, 0.15, 0.2, 0.25, and 0.3.

### Model Application Using Two Human-Relevant Datasets

The developed 19 models were applied to two datasets with organic chemicals 1) a human blood database (HBDB) comprised of anthropogenic organic chemicals identified in human blood, and 2) a subset of organic chemicals predicted to have a high exposure index to consumers, from the Swedish Product Register (SE-PR) with currently used and registered chemicals in Sweden 2018. Note that some of the compounds in HBDB and SE-PR were tested in vitro and thus appear in our training or validation sets for model training (see model predictions in the Supporting Information). A more detailed description of the two human-relevant chemical lists can be found in the Supporting Information.

## Results and Discussion

In total, 26 established or putative MIEs have been described for the TH system,^11^ and in this study, we identified and compiled 19 datasets with qualitative information for 14 MIEs. Most datasets are highly imbalanced, containing fewer active chemicals compared to inactive (Table 1). The Tox21 datasets are larger than sets retrieved from scientific peer-reviewed publications and have only ∼3% actives on average. This constitutes a challenge in in silico modeling where few methods are capable of handling such imbalanced datasets without applying over- and/or under-sampling techniques. CP was chosen as a computational framework enabling handling the imbalanced data in screening large chemical libraries and prioritization of compounds for further testing with confidence.^43^

### Model Validation

The targets of xenobiotic transformation reactions are activated by a large variety of compounds and are involved in a number of pathways.^44,45^ They have an indirect impact on the TH system regulation, and here we will refer to their corresponding models as the “general toxicity models” (Table S2) and to the others as the “thyroid-specific models” (Tables 2 and S1).

CP models were considered valid if validity for both active and inactive classes were close to the chosen confidence level (i.e., 1-SL); here, a difference of 0.01 was considered negligible.^35,37^ As seen from Table 2, all developed models are valid with an SL of 0.1 and lower, and no models show validity for both the active and inactive classes at an SL of 0.3 (Table S1). Depending on the chosen SL, the CP delivers prediction regions of different sizes. CP tends to yield more uncertain “both” predictions with a lower SL and consequently has higher validity along with lower efficiency. As we increase the SL, CP yields more single labeled predictions (active or inactive) and more predictions in the “empty” region. This pattern can be seen in all our modeling examples (Tables 2 and S1). Most CPs are conservative with validities greater than desired confidence level 1-SL. This is in particular true for the active class (Tables 2 and S1). Moreover, the models tend to produce more single-label predictions for active compounds compared to inactive at a very low SL (SL = 0.01), which could be explained by the fact that for many datasets tested the fraction of active compounds is much smaller than the fraction of inactives (Table 1), possibly due to wide range of biological reasons causing inactivity.

**Table 2:** Performance of Thyroid-Specific Conformal Prediction Models with Significance Level (SL) of 0.01, 0.1, and 0.25. nAp, nIAp, nBp, and nEp—Number of Compounds Predicted as “Active”, “Inactive”, “Both”, and “Empty” in the Validation Set; TPR—True Positive Rate; FPR—False Positive Rate; TDR(A, B)—True Discovery Rate for “Active” and “Both” Regions; NPV—Negative Predictive Value

						active		inactive		both		empty		
model	SL	validity actives	validity inactives	efficiency actives	efficiency inactives	nAp	TDR.A	nIAp	NPV	nBp	TDR.B	nEp	TPR	FPR
**TRHR**	0.01	1.00	0.99	0.10	0.01	12	0.08	0	NA	1329	0.01	0	0.10	0.01
	0.1	1.00	0.92	0.50	0.33	108	0.05	341	1.00	892	0.01	0	0.50	0.08
	0.25	0.90	0.77	1.00	0.99	296	0.03	1027	1.00	11	0.00	7	0.90	0.22
**TSHR agonist**	0.01	1.00	0.99	0.31	0.02	26	0.65	10	1.00	1242	0.03	0	0.31	0.01
	0.1	0.96	0.91	0.67	0.59	143	0.24	620	1.00	515	0.03	0	0.63	0.09
	0.25	0.81	0.75	1.00	0.98	353	0.12	901	0.99	24	0.00	0	0.81	0.25
**TSHR antagonist**	0.01	1.00	1.00	0.17	0.00	12	0.58	1	1.00	1287	0.03	0	0.17	0.00
	0.1	0.95	0.90	0.62	0.79	148	0.16	868	1.00	284	0.06	0	0.57	0.10
	0.25	0.52	0.68	0.83	0.92	232	0.13	963	0.99	0	NA	105	0.69	0.16
**DIO1**	0.01	1.00	0.99	0.12	0.02	7	0.71	3	1.00	335	0.11	0	0.12	0.01
	0.1	0.90	0.91	0.61	0.60	48	0.44	160	0.98	137	0.12	0	0.51	0.09
	0.25	0.80	0.75	0.95	0.99	107	0.29	233	0.97	5	0.40	0	0.76	0.25
**DIO2**	0.01	1.00	0.99	0.07	0.09	7	0.57	22	1.00	316	0.16	0	0.07	0.01
	0.1	0.98	0.91	0.51	0.59	53	0.51	147	0.99	145	0.19	0	0.49	0.09
	0.25	0.85	0.73	1.00	0.97	124	0.38	212	0.96	9	0.00	0	0.85	0.27
**DIO3**	0.01	1.00	0.99	0.10	0.04	8	0.75	10	1.00	327	0.16	0	0.10	0.01
	0.1	0.92	0.91	0.56	0.53	55	0.51	129	0.96	161	0.16	0	0.47	0.09
	0.25	0.83	0.77	0.78	0.88	102	0.35	197	0.95	46	0.28	0	0.61	0.23
**NIS**	0.01	1.00	1.00	0.00	0.00	0	NA	0	NA	41	0.20	0	0.00	0.00
	0.1	1.00	0.91	0.50	0.15	7	0.57	2	1.00	32	0.13	0	0.50	0.09
	0.25	1.00	0.67	0.88	0.85	18	0.39	17	1.00	6	0.17	0	0.88	0.33
**TPO**	0.01	1.00	0.99	0.12	0.06	8	0.88	7	1.00	183	0.28	0	0.12	0.01
	0.1	0.90	0.92	0.68	0.55	45	0.76	72	0.92	81	0.23	0	0.58	0.08
	0.25	0.81	0.71	0.97	0.97	87	0.53	105	0.90	6	0.33	0	0.78	0.29
**TTR**	0.01	1.00	1.00	0.00	0.00	0	NA	0	NA	45	0.40	0	0.00	0.00
	0.1	0.94	0.89	1.00	0.93	20	0.85	23	0.96	2	0.00	0	0.94	0.11
	0.25	0.72	0.44	0.89	0.74	16	0.94	20	0.95	0	NA	9	0.83	0.04
**TRß agonist**	0.01	1.00	0.99	0.29	0.01	12	0.17	0	NA	1308	0.00	0	0.29	0.01
	0.1	1.00	0.92	0.43	0.08	110	0.03	0	NA	1210	0.00	0	0.43	0.08
	0.25	0.86	0.79	0.57	0.64	280	0.01	569	1.00	471	0.01	0	0.43	0.21
**TRß antagonist**	0.01	1.00	0.99	0.11	0.01	14	0.50	3	1.00	1048	0.05	0	0.11	0.01
	0.1	0.98	0.93	0.63	0.62	105	0.37	558	1.00	402	0.06	0	0.61	0.07
	0.25	0.91	0.78	1.00	0.99	261	0.22	797	0.99	0	NA	7	0.91	0.20

To find the most suitable confidence level for model application for our in-house databases, we constructed graphs representing efficiency, TPR, FPR, and TDR.A calculated on the validation set predictions. Most of these statistical metrics follow a general trend with some exceptions. Noticeably, models built on more balanced data show higher efficiency and TDR.A (DIOs, TPO, and TTR). The deviations and worse model performance discussed further, in particular for TRHR and TRß agonist models, might be due to an extreme imbalance of the data. The datasets for these models contain only 0.73 and 0.52% active compounds, respectively (Table 1). Despite that, models increase the chances of picking active compounds among the predicted actives several times compared to the whole validation set. Despite that the CP framework can work on highly imbalanced sets, it still has its limitations, and will not commonly generate high TDR for highly imbalanced sets with a low fraction of actives.

**Figure 2:**
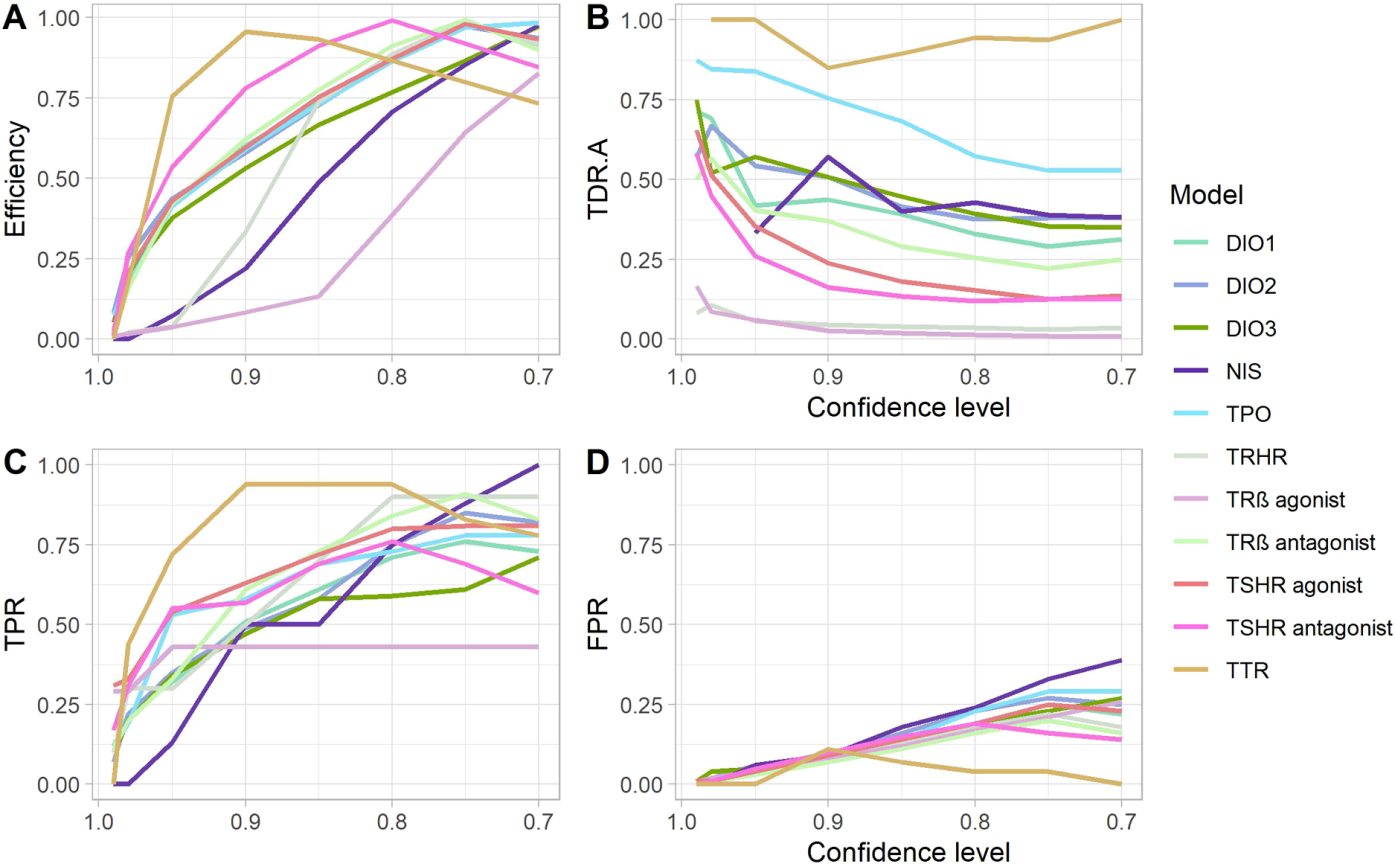
(A) Efficiency, (B) true discovery rate for the “active” region (TDR.A), (C) TPR, and (D) FPR of thyroid-specific CP models with different confidence levels.

Ideally, we would like to achieve both high TDR.A and TPR along with the majority of predictions being a singleton in a valid CP. With the high confidence setting, CP’s efficiency tends to be low. When increasing the acceptable error rate SL, more singleton predictions appear. An indication of a useful CP model would be having a peak in efficiency followed by a drop (Figures 2, S1). The efficiency estimate is negatively correlated with the confidence level and thus the most efficient CPs are found at SLs of 0.2 and 0.25 (Figures 2, S1, and Table S1). Many models give more than 50% certain singleton predictions at a confidence level of 0.9, and those that are less efficient at that level reach 60–90% efficiency at the lowest confidence level when the model is valid. High efficiency is coupled with higher TPR and lower TDR.A, which is shown in all our models (Tables 2, S1, and Figure 2).

TPR of the developed models increases with lowering confidence level (Figure 2C) with slight deviations in the TRβ agonist and NIS models, where TPR reaches the plateau below 50% (TRβ agonist) or increases constantly up to 100% in the case of NIS. For TTR and NIS inhibition models TPR in the most confident setting is zero as no compounds are predicted to be in the “active” region, but in “both” (Tables 1, S1). FPR (Figure 2D) decreases with the confidence level, except for the TTR model, which has a peak of false positives at 0.9 confidence level when the model is the most efficient.

CP models have an advantage in that the number of revealed active compounds and the experimental costs for the confirmation studies can be controlled by selecting an acceptable error rate. The decision can be done based on the purpose of the modeling activity, the desired number of compounds retrieved, and the affordable error rate of the model.

To estimate the cost of finding an active compound in the prediction regions, we calculated TDR for the “active” (TDR.A) and “both” (TDR.B) regions (Table 2). These metrics vary from model to model and depend on the chosen SL, but generally, it drops by decreasing the confidence level (Table 2, Figure 2). For the prioritization and selection of a small number of active chemicals, the highest TDR.A is searched while yielding a low FPR. The criterion is maximized for most models when selecting an SL of 0.01 (or 0.02 for some of them); however, the efficiency and TPR are very low at such high confidence settings. For larger high throughput screening purposes, even chemicals predicted in the “both” region can be of interest, if the TDR for this region is high enough. The majority of test chemicals appearing in this region are experimentally inactive (Table 2) which has been also shown in previous studies.^37^ Noticeably, TDR.B is, in general, higher for smaller datasets (DIOs, NIS, TPO, and TTR).

For the 0.9 confidence level, TDR.A varies in our models from rates exceeding 50% for TPO, DIO2, DIO3, NIS inhibition, and TTR binding models among thyroid-specific models and AHR, CAR, and PXR agonist among general models, to rates below 10% for TR-beta agonist, TRHR, and PPAR-delta. For both thyroid-specific and general MIEs, models built on Tox21 datasets show lower TDR.A as the number of false positives tends to be several times higher than the number of true positives, despite having very few actives in the datasets in most cases (Tables 1 and S1).

### Application of the Models to Two Human Relevant Datasets

The developed models were applied to two in-house databases to identify potential THDCs with no or limited information on TH system-related activity. The long-term goal is to use these lists of potential THDCs for future confirmation studies. The significance level was set to 0.1, as in this prioritization process reasonably high TDR.A and low FPR were more desirable than high efficiency. All chemicals in HBDB and SE-PR were predicted to be inside the model’s applicability domain under the setting SL = 0.1, thus the predictions include 3 regions: “active”, “inactive”, and uncertain (“both”).

**Figure 3:**
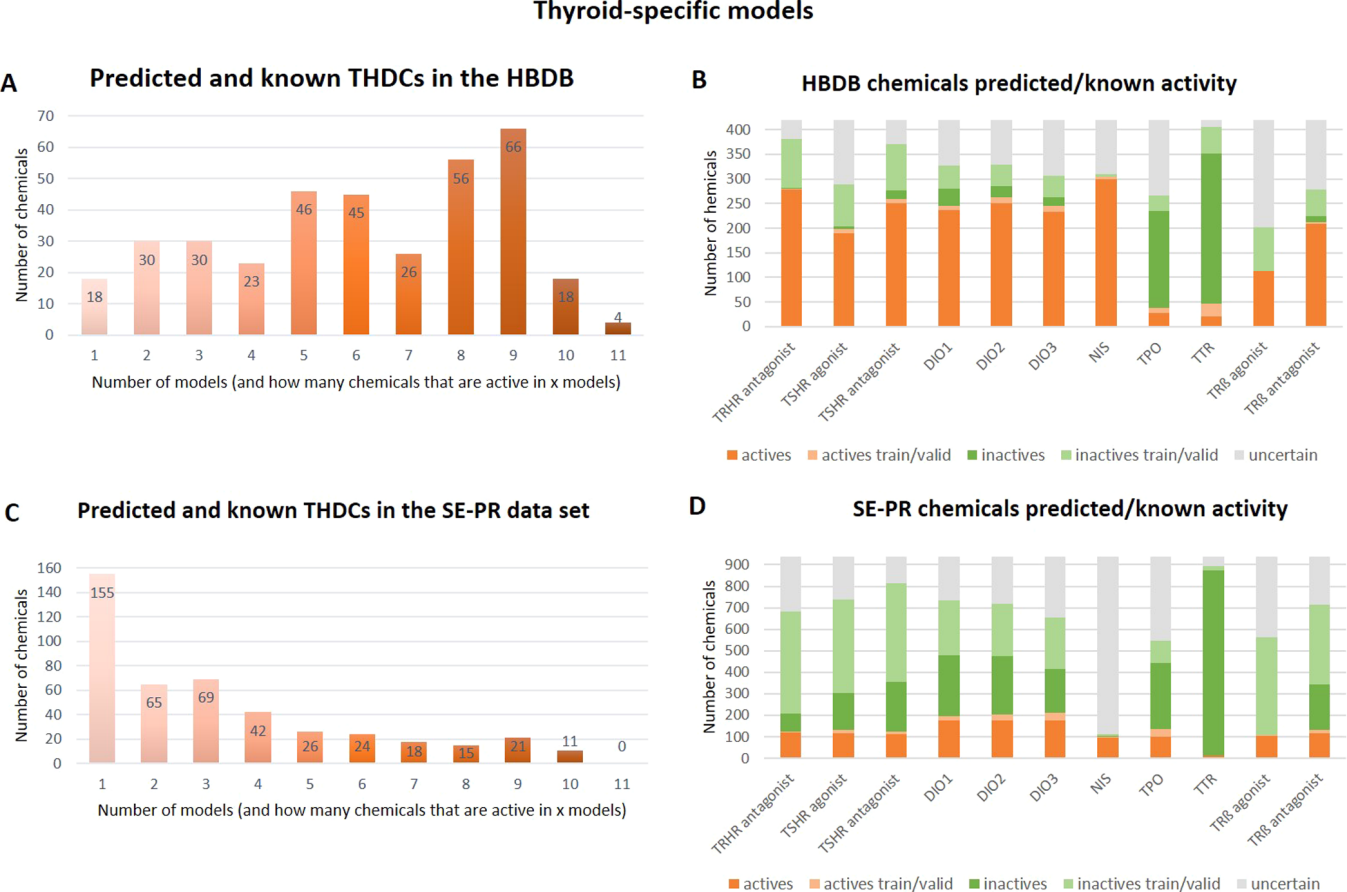
Number of chemicals predicted/known as potential THDCs per number of thyroid-specific models in the HBDB (A) and the activity/inactivity/uncertain distribution (B), and in the Swedish Product Register (SE-PR, C,D) with significance level = 0.1. Light green and light orange colors represent compounds confirmed inactive/active (known) in the model training data and dark colors represent compounds predicted as inactive/active; grey represent compounds not given certain predictions (those predicted both).

Chemicals identified in human blood tend to have potential to interfere with the TH system with 362 out of 419 (86%) chemicals predicted as active in at least one thyroid-specific model (Figure 3A,B), in comparison with the SE-PR list, where only 446 out of 937 (47%) chemicals (Figure 3C,D) were predicted active. Furthermore, the majority of the chemicals in HBDB were found active in five or more thyroid-specific models, compared to the chemicals in SE-PR, where the majority of chemicals were either not tested or predicted active by any model or active in one model only. The same pattern can be seen for the general toxicity models, where 14% of the chemicals in HBDB and 71% of the chemicals in the SE-PR dataset were not predicted active in any model (Figure S2). The details of the chemicals prediction results in each model for the two datasets HBDB and SE-PR are given in Supporting Information.

TPO and TTR models predicted remarkably fewer compounds as actives and more as inactives in HBDB than other models with the same SL. All metabolites of brominated flame retardants were predicted active TPO inhibitors and TTR binders, and 88% of phenols were predicted as TPO inhibitors. Among PCB metabolites in HBDB 75% were found to be potent TTR binders which may be explained by the chemical space covered in the training data for these models (see Materials and Methods section) and that HBDB contains around 80% of halogenated chemicals. Binding to TTR is favored by compounds having a hydroxyl group.^27,28^ Bisphenols, with two hydroxyl groups, have been predicted to be nonbinders to TTR, which could be due to the fact that bisphenol A (BPA) was tested inactive in the TTR-binding assay. On the other hand, tetrabromobisphenol A was tested active which indicates the role of the halogens in the binding to TTR.^27,28^

Four brominated flame retardant metabolites (4-OH-BDE90, 5′-OH BDE99, 6-OH-BDE137, and 6-OH-BDE99) in the HBDB were predicted active by all thyroid-specific models, and seven or eight general models. Typically, the hydroxylated metabolites of the brominated flame retardants and chlorinated biphenyls, which have structural similarities with the thyroid hormones, were predicted to be active in 10 or 11 thyroid-specific models.

In the SE-PR dataset, the most active chemicals (≥9 hits in the thyroid-specific models) are interestingly the pigments (*n* = 29 of 32). This is a group of chemicals, mainly consisting of azo or polycyclic pigments, that have to our knowledge not been studied for their endocrine-disrupting potency. Little is known about the toxicity of these chemicals but metabolites of azo-dyes are known allergens and carcinogens.^46^ Key for their performance as pigments is that they are insoluble in water and any other standard solvents, and consequently organic pigments have been predicted to be “poorly soluble particles without intrinsic toxicity potential”.^47^ Notably, none of the pigments were active in the TTR-binding model.

Many compounds from both HBDB and SE-PR that were tested or predicted as DIO inhibitors share the potential to inhibit all three DIOs. This has also been shown in the in vitro data^31^ that the models were trained on. DIOs are known to share catalytic domain properties and thus inhibition mechanisms.^48^ In total, 68% of compounds in HBDB show inhibition of any DIO, and among them, 50% are predicted to inhibit all three DIOs. Approximately 68% of PCBs and flame retardants, along with their metabolites were tested or predicted inhibitors of three DIOs. On the contrary, only 12% of pesticides and their metabolites in HBDB showed potency to inhibit all DIOs in vitro or in silico. In the SE-PR dataset, only 28% of the chemicals were predicted or measured to inhibit any DIO; however, among those compounds, 59% were tested or predicted inhibitors of all three types of DIOs.

The NIS inhibition model showed a significant difference in the number of retrieved potential active compounds in HBDB versus SE-PR when the number of predicted actives is three-fold higher in HBDB than in SE-PR. In the HBDB, 57% of 300 chemicals predicted active are expected to be truly active (Table 2, SL = 0.9). In contrast, the majority of compounds in SE-PR were not given certain predictions with a 90% confidence level (Figure 3D), which might be explained by the narrow chemical domain covered in the NIS training data (see Supporting Information).

The predicted response pattern in TH-related activities was compared and illustrated for three classes of chemicals of emerging concern, that is, phthalates, parabens, and bisphenols (Figures S3–S5). This was done to illustrate not only the complexity in responses but also to investigate the potential of chemical grouping related to disruption of the thyroid system. Within the field of mixture risk assessment, there is a need to identify groups of chemicals that exhibit similar toxicological profiles in a specific organ or system.^49^ The application of in silico models can be a first-tier approach to apply in, for example, the cumulative assessment group methodology for assessing risks related to the thyroid hormone system.^50^ The predicted expression profiles of studied MIEs related to the TH system were clearly disparate with phthalates and parabens displaying mainly inactivity, whereas bisphenols include chemicals that evoke a range of MIEs (Figures S3–S5). It is also clear that these groups of emerging chemicals are well-studied with experimental data covering several MIEs^50^ and that the models are capable of filling data gaps (see Supporting Information for prediction details). The phthalates, used as plasticizers, are known endocrine disruptors on the androgen system,^51^ but nearly no activity was predicted on the thyroid-specific pathways. The predictions are not surprising as 48% of the phthalate data in the HBDB was used in the model’s training set, among which 46% were labeled as “inactive” and only a small fraction (2%) as “active” (Figure S3A). Parabens, used as preservatives in cosmetic and pharmaceutical products, were confirmed or predicted to be inactive in thyroid-specific models (Figure S4A). Some of the phthalates and parabens, however, show confirmed or predicted activity in one or two models of general toxicity (Figures S3B and S4B).

In a recent review, it was concluded that toxicological information is not available for most BPA substitutes.^52^ In the present study, all bisphenols were confirmed or predicted active in more models than BPA, except for bisphenol S (BPS) (Figure S5). Bisphenols of special concern are bisphenol G, bisphenol M, bisphenol P, and bisphenol NP as they were predicted active in ≥9 thyroid-specific models and ≥7 general toxicity models. BPA is for example replaced with BPS in thermal paper,^53^ which can be questioned concerning the resemblance in thyroid-specific expression profiles (Figures S5A). Notably, however, BPS was predicted as less active in the general toxicity models compared to BPA (Figure S5B). When regulating chemicals, grouping has been suggested as an approach to avoid regrettable substitutions, such as in the case of BPA.^54^ This analysis has revealed similarities in hazard profiles in a chemical class but also certain dissimilarities.

### Environmental Implications

First-tier qualitative models covering the majority of known MIEs of the thyroid hormone system were developed enabling the classification of large chemical inventories with high confidence. It was also clear that for certain MIEs (TRHR, NIS, and TRβ), data are lacking both in the numbers of tested chemicals and the chemical representativity of training sets. The presented set of models can be further enriched when more data is generated, and in addition, the screening can be complemented with additional MIEs of the thyroid hormone system as data is becoming available. We also suggest using a developed framework for other MIEs of relevance for other endocrine systems. The developed CP models can be tuned depending on the purpose of the modeling activity by setting an appropriate confidence level. The application of these models showed that among currently used chemicals, fewer are overall predicted as active compared to chemicals found in human blood.

To prioritize compounds of concern for further and more detailed hazard assessment using the developed first-tier models, the number of models predicting a chemical as active was taken into consideration. Thus, we assume that a compound predicted to be in the “active” region by many thyroid-related CP models has a higher likelihood to be a cause of concern for disturbing the thyroid hormone system. By applying the models to the two selected chemical inventories, we identified chemicals of specific concern for TH disruption from both databases based on their predicted activity; 88 compounds from the HBDB and 32 from the SE-PR list were predicted to be active by at least nine CP models (Figure 3A,C; Table S4). Nearly half of the 88 compounds from the HBDB list are PCBs, flame retardants, and metabolites of these classes of chemicals. A majority of the predicted active chemicals in the SE-PR list are pigments, and 80% contain aromatic substructures. These compounds are proposed for a more detailed hazard assessment. The chemical space of the HBDB and SE-PR chemicals are shown in the Supplement along with the clustering of predicted active chemicals. Developed models are available for application on GitHub (https://github.com/Feesterra/Conformal_THS).

## Supporting Information Available

CP model performance statistics; HBDB chemical groups; prediction distributions for in-house databases (HBDB and SE-PR); predicted activity for selected chemical groups in HBDB; and HBDB and SE-PR chemicals proposed for detailed hazard assessment (PDF)
CP model training data (XLSX)
Predictions for HBDB and SE-PR compounds (XLSX)
