# Supplementary material for: In Silico Identification of Potential Thyroid Hormone System Disruptors among Chemicals in Human Serum and Chemicals with a High Exposure Index
Source: Environ Sci Technol. 2022 May 13;56(12):8363–72. doi: 10.1021/acs.est.1c07762 (PMC9228062; doi:10.1021/acs.est.1c07762)
Supplement: Supplementary file 1 — es1c07762_si_001.pdf [file es1c07762_si_001.pdf]

Supporting information for

In silico identification of potential thyroid hormone system disruptors among chemicals in human serum and chemicals with high exposure index

Elena Dracheva†, Ulf Norinder‡, Patrik Rydén¥, Josefin Engelhardt§, Jana M. Weiss§, Patrik L. Andersson†*

† Department of Chemistry, KB.E6, Linnaeus väg 6, Umeå University, SE-901 87 Umeå, Sweden

‡ Department of Computer and Systems Sciences, Stockholm University, Box 7003, SE-164 07 Kista, Sweden

¥ Department of Mathematics and Mathematical Statistics, MIT.E.351, Umeå University, SE-901 87 Umeå, Sweden

§ Department of Environmental Science, Stockholm University, SE-11418 Stockholm

* Corresponding author: elena.dracheva@umu.se, dracheva.elina@gmail.com

21 pages
4 tables
6 figures

Contents

S1. Dataset preparation S3

S2. Information on Human Blood Data Base (HBDB) and Swedish Product Register (SE-PR) lists S4

Table S1 ..... S5

Table S2 ..... S8

Figure S1 ..... S11

Table S3 ..... S12

Figure S2. ..... S13

Figure S3 ..... S14

Figure S4 ..... S15

Figure S5 ..... S16

Table S4 ..... S17

Figure S6 ..... S20

References S21

S1. Dataset preparation

Thyroperoxidase (TPO): the TPO inhibition dataset was taken from Friedman et al.[1] and in this study, 1074 ToxCast chemicals were screened in a single-concentration using the Amplex UltraRed TPO assay for their potential to inhibit TPO. In brief, the screening revealed 314 potent TPO inhibitors that elicited more than 20% decrease in maximal TPO activity. They were then divided into 3 categories; "highly selective", "low selective" and "non-selective" as defined by Friedman et al.[1] and then the non-selective compounds decreasing TPO activity by less than 22% were removed to obtain a more specific dataset with TPO inhibitors.

Iodothyronine deiodinases 1, 2, 3 (DIOs): datasets for inhibition of three deiodinases (DIO1, DIO2 and DIO3) were taken from Olker et al[2]. In this work, 1800 chemicals from the ToxCast database were screened for chemicals potency to inhibit DIO1, DIO2 and DIO3 in single-concentration experiments. The 240 potent inhibitors were tested in a concentration-response mode. Putative deiodinase inhibitors were defined as those confirmed in concentration-response screening and those showing more than 20% inhibition in the single concentration screening.

Sodium/iodide symporter (NIS). Data for NIS inhibition was taken from Wang et al[3], who screened 293 unique chemicals selected from the ToxCast Phase I library in the Radioactive Iodide Uptake (RAIU) assay. The library contains environmental contaminants, mostly pesticides and antimicrobials. After the initial single concentration testing, full dose-response experiments for 136 chemicals were performed in which 90 were eliminated due to cytotoxicity.

S2. Information on Human Blood Data Base (HBDB) and Swedish Product Register (SE-PR) lists

HBDB: The HBDB used in the current study is an in-house database described in details elsewhere[4]. It consists of 440 anthropogenic organic chemicals found in human blood world-wide, reported in studies published between 2000-2020. 419 compounds with clearly defined structures and which could be processed with RDkit were used for the prediction in this study. Pharmaceuticals, endogenous compounds and metals are not included in the HBDB and neither articles written in other languages than English or Swedish. SMILES, CAS number, and other identifiers were collected for all chemicals. The database is not considered exhaustive, as individual chemical names could have been foreseen with the general search terms used (we refer to the original publication by Engelhardt et al.[4]. for the details). The chemicals detected in blood HBDB mainly consists of nonpolar chemicals with 83% halogenated structures and 69% aromatic structures, out of which 11% are phenolic. The chemical groups in the HBDB are listed in Table S3.

SE-PR: Anyone manufacturing or importing products to Sweden have to provide information on chemicals and chemical products to the Swedish Chemicals Agency. The information is stored in the SE-PR, containing information on e.g., quantity, product category and sector of use, and if the product is available to consumers. To be able to predict the potential of the chemicals in products to reach different human and environmental media, an exposure index (EI) was developed and applied to the SE-PR[5]. The target exposure matrices in the EI includes surface water, soil, air, sewage treatment plant and human (occupational and consumer). The calculation of the index utilizes use categories, quantities, consumer availability, hazard labelling, and number of products containing the compound. An evaluation of the calculation resulted in a slightly updated calculation and is described in detail elsewhere[6]. The SE-PR dataset used here contains organic chemicals with the highest exposure index to consumers (>6), and consists of 937 individual entries.

Table S1. Performance of thyroid-specific conformal prediction models with significance levels (SL) of 0.02, 0.05, 0.15 and 0.3. nAp, nIAp, nBp, nEp – number of compounds predicted as “active”, “inactive”, “both”, and “empty”; TPR – true positive rate; FPR – false positive rate; TDR(A, B) – true discovery rate for “active” and “both” regions; NPV – negative predictive value

Model	SL	nA test*	nIA test*	validity actives	validity inactives	efficiency actives	efficiency inactives	active		inactive		both		empty	TPR	FPR
								nAp	TDR.A	nIAp	NPV	nBp	TDR.B	nEp		
**TRHR antagonists**	0.02	10	1331	1.00	0.98	0.30	0.02	28	0.11	0	NA	1313	0.01	0	0.30	0.02
	0.05	10	1331	1.00	0.96	0.30	0.04	53	0.06	0	NA	1288	0.01	0	0.30	0.04
	0.15	10	1331	0.90	0.87	0.80	0.74	179	0.04	811	1.00	351	0.01	0	0.70	0.13
	0.3	10	1331	0.90	0.65	1.00	0.92	248	0.04	982	1.00	0	NA	111	0.90	0.18
**TSHR agonist**	0.02	54	1224	1.00	0.99	0.33	0.21	35	0.51	234	1.00	1009	0.04	0	0.33	0.01
	0.05	54	1224	0.98	0.96	0.56	0.42	82	0.35	467	1.00	729	0.03	0	0.54	0.04
	0.15	54	1224	0.93	0.86	0.80	0.75	216	0.18	747	0.99	315	0.03	0	0.72	0.14
	0.3	54	1224	0.80	0.63	0.98	0.93	322	0.14	869	0.99	0	NA	87	0.81	0.23
**TSHR antagonist**	0.02	42	1258	1.00	0.99	0.31	0.27	29	0.45	323	1.00	948	0.03	0	0.31	0.01
	0.05	42	1258	0.95	0.95	0.60	0.53	88	0.26	608	1.00	604	0.03	0	0.55	0.05
	0.15	42	1258	0.86	0.85	0.83	0.91	217	0.13	969	0.99	114	0.06	0	0.69	0.15
	0.3	42	1258	0.26	0.57	0.67	0.85	195	0.13	905	1.00	0	NA	200	0.60	0.14
**DIO1**	0.02	41	304	0.98	0.99	0.24	0.19	13	0.69	55	0.98	277	0.11	0	0.22	0.01
	0.05	41	304	0.98	0.94	0.34	0.43	31	0.42	113	0.99	201	0.13	0	0.32	0.06
	0.15	41	304	0.90	0.87	0.71	0.74	64	0.39	191	0.98	90	0.13	0	0.61	0.13

Model	SL	nA test*	nIA test*	validity actives	validity inactives	efficiency actives	efficiency inactives	active		inactive		both		empty	TPR	FPR
								nAp	TDR.A	nIAp	NPV	nBp	TDR.B	nEp		
	0.3	41	304	0.61	0.63	0.88	0.92	96	0.31	220	0.97	0	NA	29	0.73	0.22
**DIO2**	0.02	55	290	1.00	0.98	0.22	0.26	18	0.67	70	1.00	257	0.17	0	0.22	0.02
	0.05	55	290	1.00	0.94	0.35	0.46	35	0.54	116	1.00	194	0.19	0	0.35	0.06
	0.15	55	290	0.95	0.84	0.64	0.74	77	0.42	174	0.98	94	0.21	0	0.58	0.16
	0.3	55	290	0.75	0.62	0.93	0.94	118	0.38	205	0.97	0	NA	22	0.82	0.25
**DIO3**	0.02	59	286	1.00	0.96	0.20	0.20	23	0.52	47	1.00	275	0.17	0	0.20	0.04
	0.05	59	286	0.95	0.95	0.39	0.37	35	0.57	95	0.97	215	0.17	0	0.34	0.05
	0.15	59	286	0.88	0.85	0.69	0.66	76	0.45	154	0.95	115	0.16	0	0.58	0.15
	0.3	59	286	0.71	0.69	0.97	0.98	120	0.35	216	0.93	3	0.33	6	0.71	0.27
**NIS**	0.02	8	33	1.00	1.00	0.00	0.00	0	NA	0	NA	41	0.20	0	0.00	0.00
	0.05	8	33	1.00	0.94	0.13	0.06	3	0.33	0	NA	38	0.18	0	0.13	0.06
	0.15	8	33	1.00	0.82	0.50	0.48	10	0.40	10	1.00	21	0.19	0	0.50	0.18
	0.3	8	33	1.00	0.61	1.00	0.97	21	0.38	19	1.00	1	0.00	0	1.00	0.39
**TPO**	0.02	59	139	0.98	0.99	0.20	0.16	13	0.85	21	0.95	164	0.29	0	0.19	0.01
	0.05	59	139	0.92	0.96	0.61	0.33	37	0.84	45	0.89	116	0.20	0	0.53	0.04
	0.15	59	139	0.90	0.86	0.80	0.71	60	0.68	85	0.93	53	0.23	0	0.69	0.14
	0.3	59	139	0.76	0.68	0.98	0.99	87	0.53	108	0.89	0	NA	3	0.78	0.29
**TTR**	0.02	18	27	1.00	1.00	0.44	0.00	8	1.00	0	NA	37	0.27	0	0.44	0.00
	0.05	18	27	0.94	1.00	0.78	0.74	13	1.00	21	0.95	11	0.36	0	0.72	0.00

Model	SL	nA test*	nIA test*	validity actives	validity inactives	efficiency actives	efficiency inactives	active		inactive		both		empty	TPR	FPR
								nAp	TDR.A	nIAp	NPV	nBp	TDR.B	nEp		
	0.15	18	27	0.94	0.70	1.00	0.89	19	0.89	23	0.96	0	NA	3	0.94	0.07
	0.3	18	27	0.61	0.33	0.83	0.67	14	1.00	19	0.95	0	NA	12	0.78	0.00
**TRß agonist**	0.02	7	1313	1.00	0.98	0.29	0.02	23	0.09	0	NA	1297	0.00	0	0.29	0.02
	0.05	7	1313	1.00	0.96	0.43	0.04	50	0.06	0	NA	1270	0.00	0	0.43	0.04
	0.15	7	1313	1.00	0.88	0.43	0.13	154	0.02	21	1.00	1145	0.00	0	0.43	0.12
	0.3	7	1313	0.57	0.74	0.86	0.83	340	0.01	752	1.00	228	0.00	0	0.43	0.26
**TRß antagonist**	0.02	64	1001	1.00	0.99	0.20	0.15	23	0.57	145	1.00	897	0.06	0	0.20	0.01
	0.05	64	1001	0.98	0.97	0.34	0.43	52	0.40	399	1.00	614	0.07	0	0.33	0.03
	0.15	64	1001	0.95	0.89	0.78	0.78	162	0.29	664	1.00	239	0.06	0	0.73	0.11
	0.3	64	1001	0.73	0.64	0.91	0.90	212	0.25	747	0.99	0	NA	106	0.83	0.16

Table S2. General CP models performance (all tested SLs). nAp, nIAp, nBp, nEp – number of compounds predicted as “active”, “inactive”, “both”, and “empty”; TPR – true positive rate; FPR – false positive rate; TDR(A, B) – true discovery rate for “active” and “both” regions; NPV – negative predictive value

Model	SL	nA test*	nIA test*	validity actives	validity inactives	efficiency actives	efficiency inactives	active		inactive		both		empty	TPR	FPR
								nAp	TDR.A	nIAp	NPV	nBp	TDR.B	nEp		
**AHR agonists**	0.01	150	1118	0.99	0.99	0.25	0.31	48	0.75	330	1.00	890	0.13	0	0.24	0.01
	0.02	150	1118	0.99	0.98	0.41	0.50	87	0.69	532	1.00	649	0.14	0	0.40	0.02
	0.05	150	1118	0.97	0.95	0.61	0.65	147	0.59	675	0.99	446	0.13	0	0.58	0.05
	0.1	150	1118	0.93	0.90	0.82	0.79	224	0.50	785	0.99	259	0.10	0	0.75	0.10
	0.15	150	1118	0.92	0.85	0.92	0.92	292	0.43	880	0.99	96	0.13	0	0.84	0.15
	0.2	150	1118	0.89	0.79	1.00	0.98	328	0.41	919	0.98	0	NA	21	0.89	0.17
	0.25	150	1118	0.73	0.67	0.90	0.91	287	0.43	861	0.99	0	NA	120	0.83	0.15
	0.3	150	1118	0.60	0.52	0.83	0.82	243	0.47	797	0.99	0	NA	228	0.77	0.11
**CAR agonist**	0.01	173	1088	1.00	0.99	0.20	0.34	42	0.81	366	1.00	853	0.16	0	0.20	0.01
	0.02	173	1088	0.98	0.98	0.35	0.45	74	0.77	476	0.99	711	0.16	0	0.33	0.02
	0.05	173	1088	0.94	0.96	0.60	0.64	133	0.70	664	0.98	464	0.15	0	0.54	0.04
	0.1	173	1088	0.89	0.91	0.82	0.81	223	0.55	803	0.98	235	0.13	0	0.71	0.09
	0.15	173	1088	0.84	0.86	0.93	0.94	283	0.47	903	0.97	75	0.16	0	0.77	0.14
	0.2	173	1088	0.79	0.77	0.98	0.96	306	0.45	911	0.97	0	NA	44	0.80	0.15
	0.25	173	1088	0.64	0.61	0.88	0.87	260	0.50	836	0.97	0	NA	165	0.75	0.12
	0.3	173	1088	0.54	0.48	0.82	0.79	226	0.55	771	0.98	0	NA	264	0.72	0.09
**CAR antagonist**	0.01	29	936	1.00	0.99	0.14	0.01	11	0.36	0	NA	954	0.03	0	0.14	0.01
	0.02	29	936	1.00	0.99	0.21	0.01	16	0.38	0	NA	949	0.02	0	0.21	0.01
	0.05	29	936	0.97	0.96	0.41	0.22	46	0.24	174	0.99	745	0.02	0	0.38	0.04
	0.1	29	936	0.93	0.92	0.59	0.53	94	0.16	420	1.00	451	0.03	0	0.52	0.08
	0.15	29	936	0.90	0.88	0.72	0.69	130	0.14	536	0.99	299	0.03	0	0.62	0.12
	0.2	29	936	0.76	0.83	0.90	0.82	181	0.10	617	0.99	167	0.02	0	0.66	0.17
	0.25	29	936	0.69	0.78	1.00	0.96	230	0.09	701	0.99	34	0.00	0	0.69	0.22
	0.3	29	936	0.69	0.62	1.00	0.91	203	0.10	676	0.99	0	NA	86	0.69	0.20
**PPAR-delta agonist**	0.01	17	1192	1.00	0.98	0.12	0.02	21	0.10	0	NA	1188	0.01	0	0.12	0.02
	0.02	17	1192	1.00	0.97	0.12	0.03	37	0.05	0	NA	1172	0.01	0	0.12	0.03

Model	SL	nA test*	nIA test*	validity actives	validity inactives	efficiency actives	efficiency inactives	active		inactive		both		empty	TPR	FPR
								nAp	TDR.A	nIAp	NPV	nBp	TDR.B	nEp		
	0.05	17	1192	1.00	0.95	0.35	0.11	70	0.09	67	1.00	1072	0.01	0	0.35	0.05
	0.1	17	1192	0.94	0.90	0.59	0.55	131	0.07	530	1.00	548	0.01	0	0.53	0.10
	0.15	17	1192	0.94	0.84	0.71	0.74	199	0.06	700	1.00	310	0.02	0	0.65	0.16
	0.2	17	1192	0.88	0.80	0.82	0.88	256	0.05	807	1.00	146	0.02	0	0.71	0.20
	0.25	17	1192	0.71	0.74	0.94	0.98	306	0.04	882	1.00	12	0.00	9	0.76	0.25
	0.3	17	1192	0.59	0.58	0.88	0.89	250	0.05	824	1.00	0	NA	135	0.71	0.20
**PPARD antagonist**	0.01	13	1151	1.00	0.99	0.23	0.01	9	0.33	0	NA	1155	0.01	0	0.23	0.01
	0.02	13	1151	1.00	0.99	0.31	0.01	17	0.24	0	NA	1147	0.01	0	0.31	0.01
	0.05	13	1151	1.00	0.95	0.31	0.05	57	0.07	0	NA	1107	0.01	0	0.31	0.05
	0.1	13	1151	1.00	0.90	0.54	0.23	118	0.06	148	1.00	898	0.01	0	0.54	0.10
	0.15	13	1151	1.00	0.85	0.69	0.53	183	0.05	435	1.00	546	0.01	0	0.69	0.15
	0.2	13	1151	0.92	0.80	0.85	0.68	241	0.04	553	1.00	370	0.01	0	0.77	0.20
	0.25	13	1151	0.92	0.75	0.92	0.82	301	0.04	654	1.00	209	0.00	0	0.85	0.25
	0.3	13	1151	0.92	0.71	1.00	0.94	350	0.03	743	1.00	71	0.00	0	0.92	0.29
**PPARG agonist**	0.01	39	1222	1.00	0.99	0.18	0.01	16	0.44	0	NA	1245	0.03	0	0.18	0.01
	0.02	39	1222	1.00	0.98	0.28	0.03	31	0.35	15	1.00	1215	0.02	0	0.28	0.02
	0.05	39	1222	1.00	0.96	0.51	0.23	72	0.28	228	1.00	961	0.02	0	0.51	0.04
	0.1	39	1222	1.00	0.91	0.59	0.47	128	0.18	467	1.00	666	0.02	0	0.59	0.09
	0.15	39	1222	1.00	0.86	0.67	0.64	197	0.13	614	1.00	450	0.03	0	0.67	0.14
	0.2	39	1222	0.95	0.82	0.79	0.80	251	0.12	756	1.00	254	0.03	0	0.74	0.18
	0.25	39	1222	0.85	0.77	0.92	0.94	316	0.09	865	0.99	80	0.04	0	0.77	0.23
	0.3	39	1222	0.69	0.68	0.92	0.97	333	0.09	883	0.99	1	0.00	44	0.77	0.25
**PPARG antagonist**	0.01	69	1057	1.00	0.99	0.19	0.06	26	0.50	54	1.00	1046	0.05	0	0.19	0.01
	0.02	69	1057	1.00	0.98	0.20	0.20	37	0.38	190	1.00	899	0.06	0	0.20	0.02
	0.05	69	1057	0.97	0.95	0.33	0.42	70	0.30	396	0.99	660	0.07	0	0.30	0.05
	0.1	69	1057	0.93	0.90	0.54	0.64	133	0.24	582	0.99	411	0.08	0	0.46	0.10
	0.15	69	1057	0.83	0.86	0.78	0.79	195	0.22	689	0.98	242	0.06	0	0.61	0.14
	0.2	69	1057	0.77	0.81	0.94	0.92	253	0.19	781	0.98	92	0.04	0	0.71	0.19
	0.25	69	1057	0.70	0.74	0.97	0.97	277	0.18	820	0.98	1	0.00	28	0.72	0.21
	0.3	69	1057	0.62	0.57	0.93	0.87	232	0.21	755	0.98	0	NA	139	0.70	0.17
**PXR agonist**	0.01	301	897	0.99	0.99	0.19	0.28	62	0.87	244	0.99	892	0.27	0	0.18	0.01

Model	SL	nA test*	nIA test*	validity actives	validity inactives	efficiency actives	efficiency inactives	active		inactive		both		empty	TPR	FPR
								nAp	TDR.A	nIAp	NPV	nBp	TDR.B	nEp		
	0.02	301	897	0.98	0.99	0.32	0.37	105	0.88	327	0.98	766	0.27	0	0.31	0.01
	0.05	301	897	0.95	0.96	0.53	0.58	181	0.81	503	0.97	514	0.27	0	0.49	0.04
	0.1	301	897	0.90	0.90	0.80	0.82	298	0.71	679	0.96	221	0.28	0	0.70	0.10
	0.15	301	897	0.84	0.85	0.96	0.95	375	0.64	763	0.94	60	0.20	0	0.80	0.15
	0.2	301	897	0.76	0.73	0.96	0.94	376	0.64	755	0.94	0	NA	67	0.80	0.15
	0.25	301	897	0.59	0.59	0.84	0.86	332	0.67	691	0.96	0	NA	175	0.74	0.12
	0.3	301	897	0.47	0.46	0.77	0.78	295	0.72	636	0.97	0	NA	267	0.70	0.09

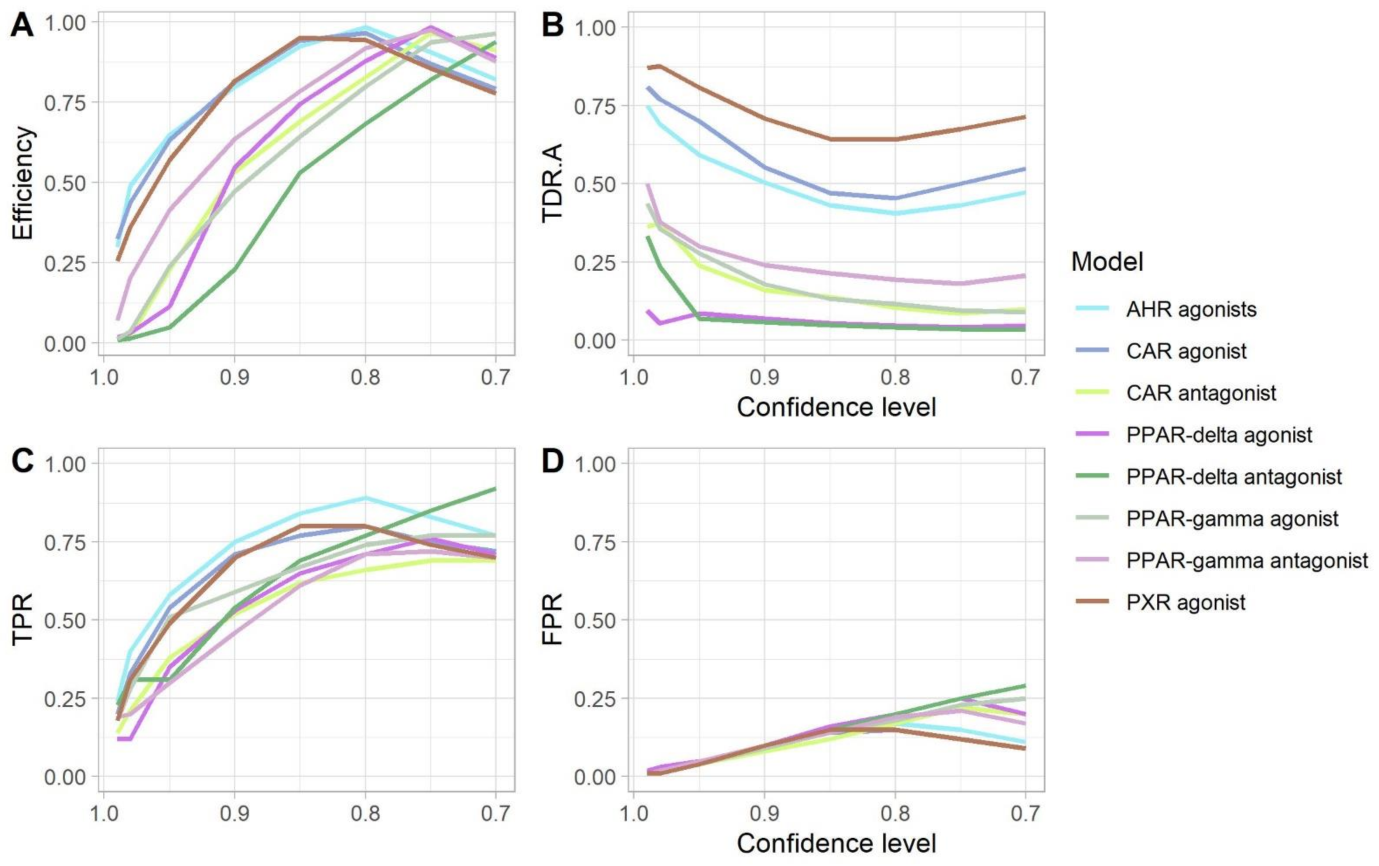

Figure S1. Efficiency, true discovery rate of "active" prediction region (TDR.A), true positive rate (TPR) and false positive rate (FPR) of general toxicity models

Table S3. Chemical groups present in the database of chemicals found in human blood (HBDB). PCB – polychlorinated biphenyl; PFAS – Per- and polyfluoroalkyl substances; PAH – polycyclic aromatic hydrocarbon

**Compound group**	**Number of compounds**
PCBs	165
PFAS	41
Pesticides	40
Flame retardants	33
Dioxin and Furans	21
Phenols	17
PAHs	14
Industrial bulk chemicals	13
PCB metabolites	12
Pesticide metabolites	10
Brominated flame retardant metabolites	9
UV-filters	9
Phthalate metabolites	7
Phthalates	7
Halogenated alkanes	5
Parabens	4
Personal care products	4
Flame retardant metabolites	3
Tobacco smoke	2
UV-filter metabolites	2
Personal care product metabolites	1

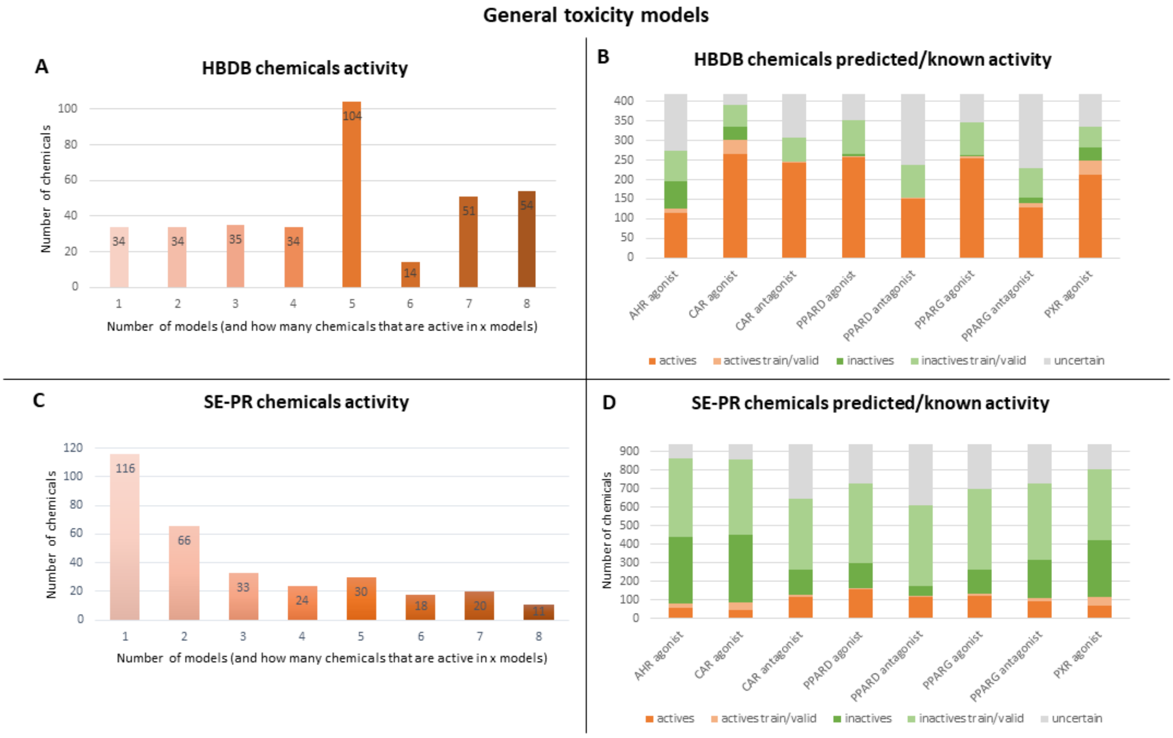

Figure S2. The number of chemicals predicted/known to potentially disrupt thyroid hormone system via general toxicity MIEs in the Human Blood database (HBDB, A) and the activity distribution (B); and in the Swedish Product Register (SE-PR, C, D) with SL=0.1. Light green and light orange colors represent compounds confirmed inactive/active (known) in the model training data, dark colors represent the newly predicted chemicals and gray color represent chemicals not given certain predictions (those predicted both or empty)

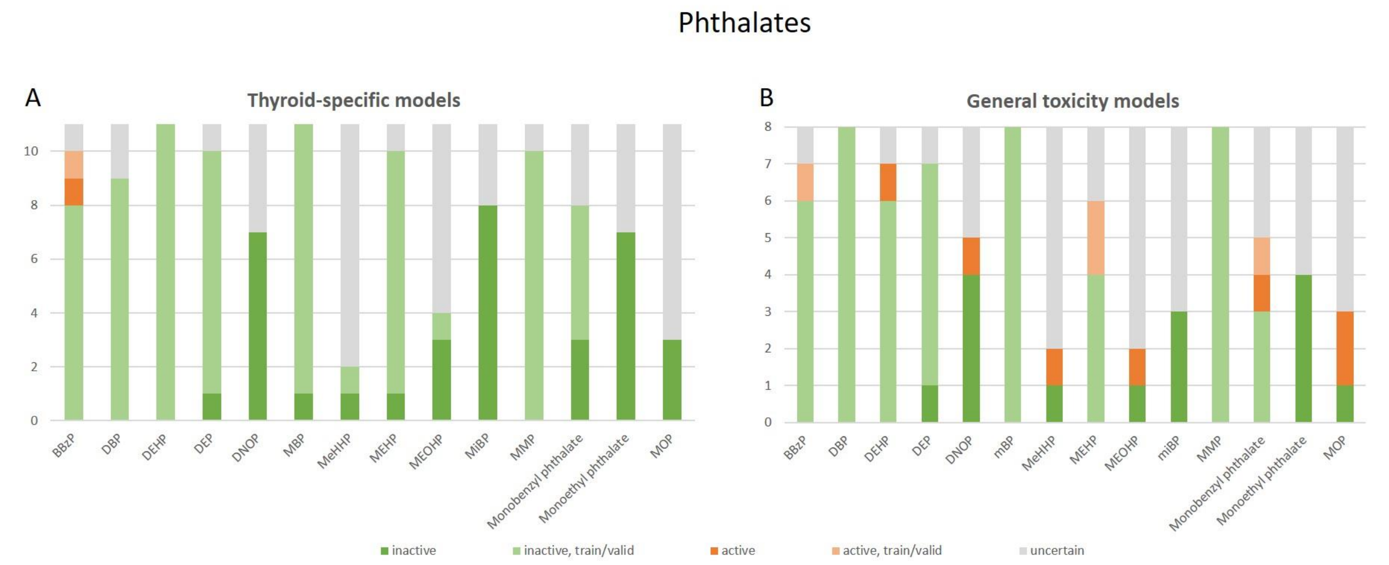

Figure S3. The number of thyroid-specific models (A) and general toxicity models (B) among phthalates found in the HBDB that have been predicted/known active/inactive. Light green and light orange colors represent compounds confirmed inactive/active in the model training data, dark colors represent the newly predicted chemicals and grey color represent chemical not given certain prediction (chemical predicted both or empty). BBzP – Benzyl butyl phthalate; DBP – Dibutyl phthalate; DEHP – Di(2-ethylhexyl) phthalate; DEP – Diethyl phthalate; DNOP – Di(2-ethylhexyl) phthalate; MBP – Monobutyl phthalate; MeHHP – Mono-2-ethyl-5-hydroxyhexyl phthalate; MEHP – Mono(2-ethylhexyl) phthalate; MEOHP – Mono(2-ethyl-5-oxohexyl)phthalate; MiBP – Isobutyl hydrogen phthalate; MMP – Monomethyl phthalate; MOP – Mono-n-octyl phthalate

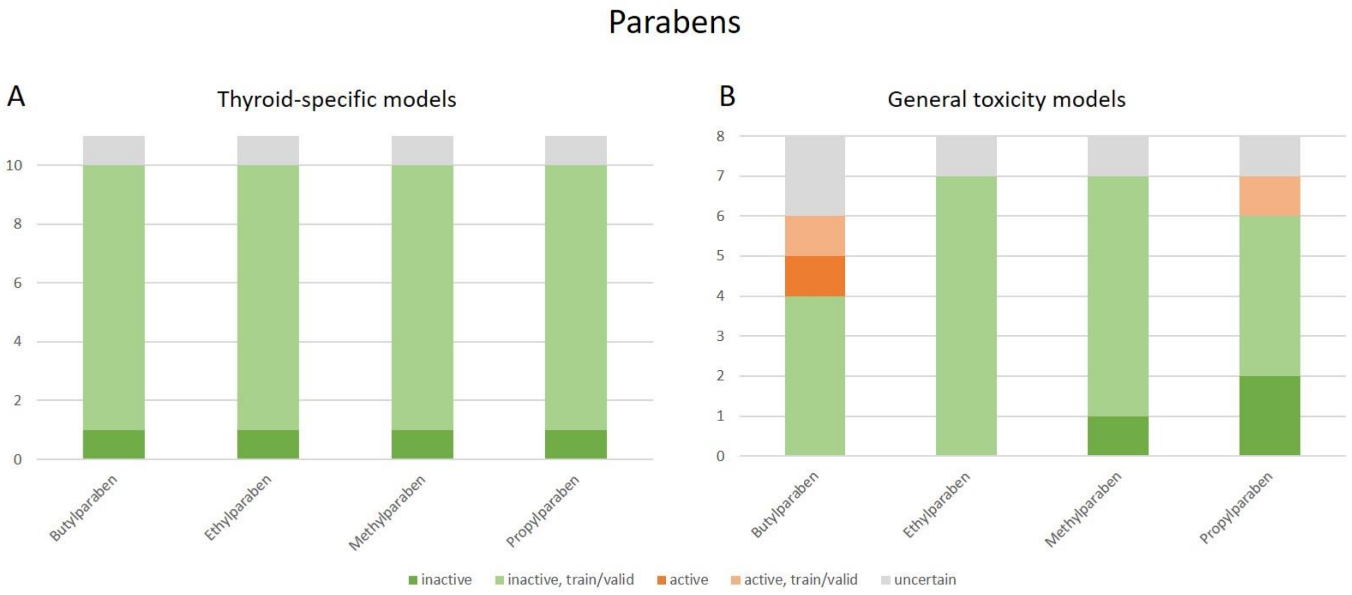

Figure S4. The number of thyroid-specific models (A) and general toxicity models (B) among parabens in the HBDB that have been predicted/known active/inactive. Light green and light orange colors represent compounds confirmed inactive/active in the model training data, dark colors represent the newly predicted chemicals and grey color represent chemical not given certain prediction (chemical predicted *both* or *empty*)

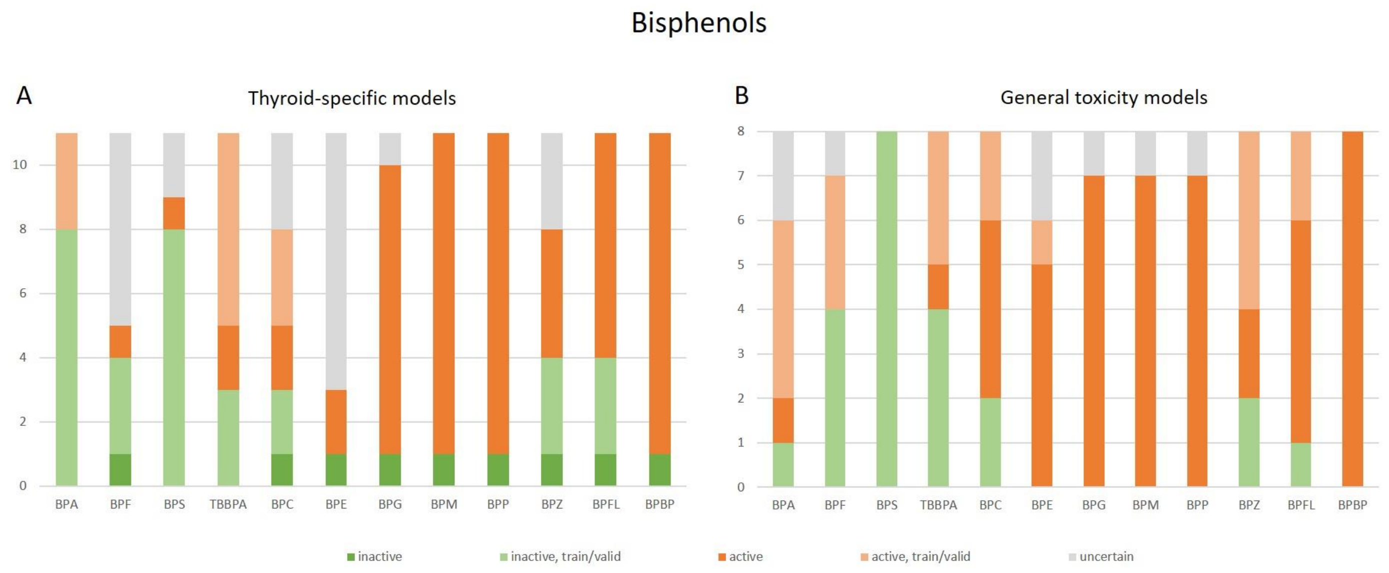

Figure S5. The number of thyroid-specific models (A) and general toxicity models (B) that bisphenols identified in the HBDB has been predicted/known active/inactive in. Light green and light orange colors represent compounds confirmed inactive/active in the model training, dark colors represent the newly predicted chemicals and grey represents chemicals not given certain prediction (chemicals predicted *both* or *empty*). BPA – bisphenol A; BPF – bisphenol F; BPS – bisphenol S; TBBPA – tetrabromobisphenol A; BPC – bisphenol C; BPE – bisphenol E; BPG – bisphenol G; BPM – bisphenol M; BPP – bisphenol P; BPZ – bisphenol Z; BPFL – bisphenol Fl; BPBP – bisphenol BP

Table S4. Compounds from the HBDB and the SE-PR list that were predicted to be active by at least 9 thyroid-specific CP models

Dataset	CAS	DTXSID	Name
HBDB	923925-51-3	DTXSID301009365	2,4-dibromo-5-(2,4,5-tribromophenoxy)phenol
HBDB	297742-10-0	DTXSID40616008	2,3,5-Tribromo-6-(2,4-dibromophenoxy)phenol
HBDB	1050642-07-3	DTXSID10903992	2,3,5-Tribromo-4-(2,4-dibromophenoxy)phenol
HBDB	111863-67-3	DTXSID50433647	CHEMBL362761
HBDB	602326-22-7	DTXSID90797013	2,3-Dibromo-4-(2,4-dibromophenoxy)phenol
HBDB	602326-23-8	DTXSID60454863	Phenol, 2,5-dibromo-4-(2,4-dibromophenoxy)-
HBDB	79755-43-4	DTXSID60229856	3,5-Dibromo-2-(2,4-dibromophenoxy)phenol
HBDB	80246-25-9	DTXSID50903991	2,4-Dibromo-6-(2,4-dibromophenoxy)phenol
HBDB	13595-25-0	DTXSID7065548	Phenol, 4,4'-[1,3-phenylenebis(1-methylethylidene)]bis-
HBDB	2167-51-3	DTXSID0058693	Bisphenol P
HBDB	149589-59-3	DTXSID50904125	2,2',3',4,4',5-Hexachloro[1,1'-biphenyl]-3-ol
HBDB	158076-69-8	DTXSID00166372	2,2',3',4,4',5,5'-Heptachloro-3-biphenylol
HBDB	158076-62-1	DTXSID10166368	(1,1'-Biphenyl)-4-ol, 2,2',3,3',4',5-hexachloro-
HBDB	145413-90-7	DTXSID60163004	(1,1'-Biphenyl)-4-ol, 2,2',3,4',5,5'-hexachloro-
HBDB	158076-68-7	DTXSID40166371	(1,1'-Biphenyl)-4-ol, 2,2',3,4',5,5',6-heptachloro-
HBDB	54284-55-8	DTXSID90202637	(1,1'-Biphenyl)-3-ol, 2,2',4,4',5,5'-hexachloro-
HBDB	158076-64-3	DTXSID80166370	2,2',3',4,5,5',6'-heptachloro[1,1'-biphenyl]-3-ol
HBDB	190317-24-9	DTXSID901009363	2,3,3',4',5,5',6-heptachloro[1,1'-biphenyl]-4-ol
HBDB	37853-59-1	DTXSID1024627	1,2-Bis(2,4,6-tribromophenoxy)ethane
HBDB	1844-01-5	DTXSID70283308	4,4'-Dihydroxytetraphenylmethane
HBDB	943913-15-3	DTXSID20873415	6:2/8:2 Fluorotelomer phosphate diester
HBDB	1895-26-7	DTXSID30172360	12:2 Fluorotelomer phosphate diester
HBDB	29082-74-4	DTXSID2021074	Octachlorostyrene
HBDB	189084-64-8	DTXSID4052689	2,2',4,4',6-Pentabromodiphenyl ether
HBDB	366791-32-4	DTXSID50573491	1,2,3-Tribromo-5-(3,4-dibromophenoxy)benzene
HBDB	182677-30-1	DTXSID60872265	2,2',3,4,4',5'-Hexabromodiphenyl Ether
HBDB	63387-28-0	DTXSID30881107	1,2,3,4,5-Pentabromo-6-(2,3,4,5-tetrabromophenoxy)benzene
HBDB	437701-79-6	DTXSID30451985	BDE-207
HBDB	437701-78-5	DTXSID40556652	2,2',3,3',4,5,5',6,6'-nonabromodiphenyl ether
HBDB	189084-62-6	DTXSID90873922	2,3',4',6-Tetrabromodiphenyl Ether
HBDB	93703-48-1	DTXSID80877030	BDE-77
HBDB	182346-21-0	DTXSID4052685	2,2',3,4,4'-Pentabromodiphenyl ether
HBDB	60348-60-9	DTXSID9030048	2,2',4,4',5-Pentabromodiphenyl ether
HBDB	84852-53-9	DTXSID2052732	1,1'-Oxybis[2,3,4,5,6-pentabromobenzene]
HBDB	35065-30-6	DTXSID2073481	2,2',3,3',4,4',5-Heptachlorobiphenyl
HBDB	52663-71-5	DTXSID4073540	2,2',3,3',4,4',6-Heptachlorobiphenyl
HBDB	52663-74-8	DTXSID7074167	2,2',3,3',4,5,5'-Heptachlorobiphenyl
HBDB	38411-25-5	DTXSID4074142	2,2',3,3',4,5,6'-Heptachlorobiphenyl
HBDB	40186-70-7	DTXSID9074147	2,2',3,3',4,5',6-Heptachlorobiphenyl
HBDB	52663-70-4	DTXSID2074164	2,2',3,3',4,5',6'-Heptachlorobiphenyl
HBDB	52663-67-9	DTXSID2074162	2,2',3,3',5,5',6-Heptachlorobiphenyl
HBDB	52663-64-6	DTXSID0073538	2,2',3,3',5,6,6'-Heptachlorobiphenyl

HBDB	35065-29-3	DTXSID6038299	2,2',3,4,4',5,5'-Heptachlorobiphenyl
HBDB	74472-47-2	DTXSID8074235	2,2',3,4,4',5,6-Heptachlorobiphenyl
HBDB	60145-23-5	DTXSID9074191	2,2',3,4,4',5,6'-Heptachlorobiphenyl
HBDB	52663-69-1	DTXSID7074163	2,2',3,4,4',5',6-Heptachlorobiphenyl
HBDB	74472-48-3	DTXSID3074236	2,2',3,4,4',6,6'-Heptachlorobiphenyl
HBDB	39635-31-9	DTXSID4074144	2,3,3',4,4',5,5'-Heptachlorobiphenyl
HBDB	41411-64-7	DTXSID3074151	2,3,3',4,4',5,6-Heptachlorobiphenyl
HBDB	74472-50-7	DTXSID3074238	2,3,3',4,4',5',6-Heptachlorobiphenyl
HBDB	69782-91-8	DTXSID30867845	2,3,3',4',5,5',6-Heptachlorobiphenyl
HBDB	52663-78-2	DTXSID1074171	2,2',3,3',4,4',5,6-Octachlorobiphenyl
HBDB	127-54-8	DTXSID30281401	2,2-Bis(4-hydroxy-3-isopropylphenyl)propane
HBDB	35822-46-9	DTXSID1052034	1,2,3,4,6,7,8-Heptachlorodibenzodioxin
HBDB	67562-39-4	DTXSID8052350	1,2,3,4,6,7,8-Heptachlorodibenzo[b,d]furan
HBDB	55673-89-7	DTXSID9052216	1,2,3,4,7,8,9-Heptachlorodibenzofuran
HBDB	57117-44-9	DTXSID2069155	1,2,3,6,7,8-Hexachlorodibenzofuran
HBDB	72918-21-9	DTXSID9052470	1,2,3,7,8,9-Hexachlorodibenzo[b,d]furan
HBDB	60851-34-5	DTXSID3052276	2,3,4,6,7,8-Hexachlorodibenzo[b,d]furan
HBDB	116806-76-9	DTXSID60151527	1,1'-Biphenyl, 2,2',3,4',5',6-hexachloro-4-(methylsulfonyl)-
HBDB	207122-15-4	DTXSID3052692	2,2',4,4',5,6'-Hexabromodiphenyl ether
HBDB	35854-94-5	DTXSID10873929	2,2',4,4',6,6'-Hexabromodiphenyl Ether
HBDB	207122-16-5	DTXSID8052693	2,2',3,4,4',5',6-Heptabromodiphenyl ether
HBDB	446255-39-6	DTXSID3074789	BDE-196
HBDB	117964-21-3	DTXSID9074775	BDE-197
HBDB	337513-72-1	DTXSID7074749	BDE-203
HBDB	3268-87-9	DTXSID4025799	Octachlorodibenzo-p-dioxin
HBDB	39001-02-0	DTXSID3052062	Octachlorodibenzofuran
HBDB	35694-08-7	DTXSID5074139	2,2',3,3',4,4',5,5'-Octachlorobiphenyl
HBDB	42740-50-1	DTXSID3074157	2,2',3,3',4,4',5,6'-Octachlorobiphenyl
HBDB	33091-17-7	DTXSID0074134	2,2',3,3',4,4',6,6'-Octachlorobiphenyl
HBDB	68194-17-2	DTXSID1074204	2,2',3,3',4,5,5',6-Octachlorobiphenyl
HBDB	52663-75-9	DTXSID2074168	2,2',3,3',4,5,5',6'-Octachlorobiphenyl
HBDB	52663-73-7	DTXSID2074166	2,2',3,3',4,5,6,6'-Octachlorobiphenyl
HBDB	40186-71-8	DTXSID4074148	2,2',3,3',4,5',6,6'-Octachlorobiphenyl
HBDB	2136-99-4	DTXSID0074132	2,2',3,3',5,5',6,6'-Octachlorobiphenyl
HBDB	52663-76-0	DTXSID7074169	2,2',3,4,4',5,5',6-Octachlorobiphenyl
HBDB	74472-53-0	DTXSID2074241	2,3,3',4,4',5,5',6-Octachlorobiphenyl
HBDB	40186-72-9	DTXSID50865989	2,2',3,3',4,4',5,5',6-Nonachlorobiphenyl
HBDB	52663-79-3	DTXSID6074172	2,2',3,3',4,4',5,6,6'-Nonachlorobiphenyl
HBDB	52663-77-1	DTXSID6074170	2,2',3,3',4,5,5',6,6'-Nonachlorobiphenyl
HBDB	2051-24-3	DTXSID4047541	Decachlorobiphenyl
HBDB	7426-07-5	DTXSID90225265	2,3,4,5-tetrachloro-6-hydroxy-N-phenylbenzamide
HBDB	24949-31-3	DTXSID00903986	2,6-Dibromo-3-(2,4-dibromophenoxy)phenol
HBDB	602326-21-6	NA	4'-OH-BDE 17
HBDB	1578186-46-5	NA	6:2/14:2 diPAP
HBDB	52969-11-4	DTXSID70165175	2,3,6-Trichloro-4-(3,4-dichlorophenyl)phenol
HBDB	70648-26-9	DTXSID6029915	1,2,3,4,7,8-Hexachlorodibenzofuran
SE-PR	88949-33-1	DTXSID20893830	Pigment Red 264

SE-PR	1709-70-2	DTXSID6027428	Ionox 330
SE-PR	6706-82-7	DTXSID50879809	Solvent Yellow 29
SE-PR	67990-27-6	DTXSID5052383	C.I.Solvent Yellow
SE-PR	68259-05-2	DTXSID00887313	Benzoic acid, 3,3'-[(2,5-dimethyl-1,4-phenylene)bis[iminocarbonyl(2-hydroxy-3,1-naphthalenediyl)-2,1-diazenediyl]]bis[4-methyl-, 1,1'-bis(2-chloroethyl) ester
SE-PR	81-77-6	DTXSID2026280	Vat Blue 4
SE-PR	3520-72-7	DTXSID6052031	Benzidine orange
SE-PR	5590-18-1	DTXSID4052219	3,3'-(1,4-Phenylenediimino)bis(4,5,6,7-tetrachloro-1H-isoindol-1-one)
SE-PR	68310-04-3	DTXSID1052406	2-[(4-Dodecylphenyl)azo]-4-(2,4-xylylazo)resorcinol
SE-PR	65087-00-5	DTXSID0052334	2,4-Bis[(4-dodecylphenyl)azo]resorcinol
SE-PR	125-20-2	DTXSID2051633	3,3-Bis(4-hydroxy-5-isopropyl-o-tolyl)phthalide
SE-PR	980-26-7	DTXSID2052655	C.I. Pigment Red 122
SE-PR	4151-51-3	DTXSID9063320	Tris(4-isocyanatophenyl) thiophosphate
SE-PR	1745-89-7	DTXSID1061942	4,4'-Isopropylidenebis(2-allylphenol)
SE-PR	355-49-7	DTXSID40188997	Perfluorohexadecane
SE-PR	6358-30-1	DTXSID2036293	8,18-Dichloro-5,15-diethyl-5,15-dihydrodiindolo[3,2-b:3′,2′-m]triphenodioxazine
SE-PR	4378-61-4	DTXSID8044674	C.I. Vat Orange 3
SE-PR	15890-25-2	DTXSID5027776	Antimony diamyldithiocarbamate
SE-PR	5567-15-7	DTXSID1021453	C.I. Pigment Yellow 83
SE-PR	6358-85-6	DTXSID1021451	C.I. Pigment Yellow 12
SE-PR	4424-06-0	DTXSID8025913	C.I. Pigment Orange 43
SE-PR	13001-39-3	DTXSID4065317	Benzonitrile, 2,2'-(1,4-phenylenedi-2,1-ethenediyl)bis-
SE-PR	28983-56-4	DTXSID90889705	Methyl Blue
SE-PR	5521-31-3	DTXSID9029273	C.I. Pigment Red 179
SE-PR	128-80-3	DTXSID9044376	D & C Green No. 6
SE-PR	2786-76-7	DTXSID7029243	C.I. Pigment Red 170
SE-PR	56358-10-2	DTXSID30866555	2-Naphthalenamine, N-(2-ethylhexyl)-1-[2-[3-methyl-4-[2-(3-methylphenyl)diazenyl]phenyl]diazenyl]-
SE-PR	6428-31-5	DTXSID3064356	C.I. Direct Black 19, disodium salt
SE-PR	30125-47-4	DTXSID2067539	C.I. Pigment Yellow 138
SE-PR	6417-50-1	DTXSID4064345	1,4-Benzenedicarboxamide, N,N'-bis[5-(benzoylamino)-9,10-dihydro-9,10-dioxo-1-anthracenyl]-
SE-PR	59656-20-1	DTXSID9069344	1,3,4-Thiadiazole, 2,5-bis(tert-dodecyldithio)-
SE-PR	4051-63-2	DTXSID2052079	Pigment Red 177

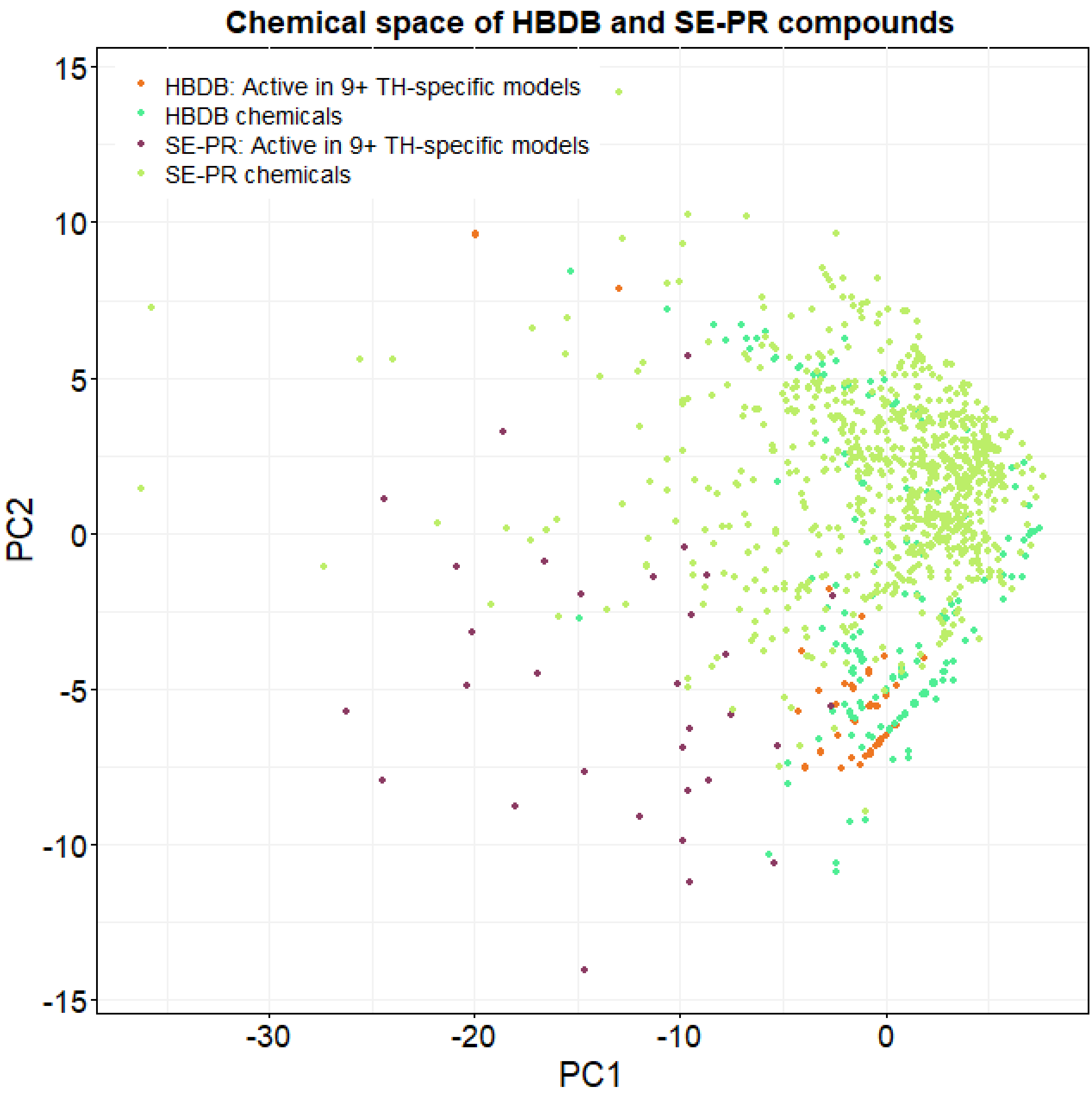

Figure S6. Chemical space of HBDB and SE-PR investigated with principal component analysis (PCA). Dimensionality is reduced to 2 dimensions explaining 35% of the total variation in the data. Compounds from HBDB and SE-PR lists predicted to be active with 90% confidence level in at least 9 thyroid-specific models are shown as orange and violet diamonds, respectively. All other compounds from the lists are shown in green and olive. Notably the most predicted active chemicals from both sets are dissimilar, HBDB actives (orange diamonds) are forming a cluster with a couple of exceptions. Most active compounds from SE-PR (violet diamonds) in general tend to separate from the rest SE-PR compounds (light-green circles)

References

1. Paul Friedman, K.; Watt, E. D.; Hornung, M. W.; Hedge, J. M.; Judson, R. S.; Crofton, K. M.; Houck, K. A.; Simmons, S. O., Tiered High-Throughput Screening Approach to Identify Thyroperoxidase Inhibitors Within the ToxCast Phase I and II Chemical Libraries. *Toxicol Sci* **2016,** *151*, (1), 160-80.
2. Olker, J. H.; Korte, J. J.; Denny, J. S.; Hartig, P. C.; Cardon, M. C.; Knutsen, C. N.; Kent, P. M.; Christensen, J. P.; Degitz, S. J.; Hornung, M. W., Screening the ToxCast Phase 1, Phase 2, and e1k Chemical Libraries for Inhibitors of Iodothyronine Deiodinases. *Toxicol Sci* **2019,** *168*, (2), 430-442.
3. Wang, J.; Hallinger, D. R.; Murr, A. S.; Buckalew, A. R.; Simmons, S. O.; Laws, S. C.; Stoker, T. E., High-Throughput Screening and Quantitative Chemical Ranking for Sodium-Iodide Symporter Inhibitors in ToxCast Phase I Chemical Library. *Environ Sci Technol* **2018,** *52*, (9), 5417-5426.
4. Engelhardt, A. J. N., K.; Weiss, J. M., Anthropogenic organic contaminants analysed in human blood and cumulative risk. In Submitted to Exposure and Health, To be published.
5. Fischer, S.; Almkvist, s.; Karlsson, E.; kerblom, M. *Preparation of a Product Register based ExposureIndex : Swedish Chemicals Inspectorate June 2006 (original version in Swedish, March 2005)*; Kemi: 2006; p 67.
6. Undeman, E.; Fischer, S.; McLachlan, M. S., Evaluation of a novel high throughput screening tool for relative emissions of industrial chemicals used in chemical products. *Chemosphere* **2011,** *82*, (7), 996-1001.
